# Rapid and accurate classification of mung bean seeds based on HPMobileNet

**DOI:** 10.3389/fpls.2024.1474906

**Published:** 2025-02-13

**Authors:** Shaozhong Song, Zhenyang Chen, Helong Yu, Mingxuan Xue, Junling Liu

**Affiliations:** ^1^ School of Data Science and Artificial Intelligence, Jilin Engineering Normal University, Changchun, China; ^2^ Smart Agriculture Research Institute, Jilin Agricultural University, Changchun, China; ^3^ College of Information Technology, Jilin Agricultural University, Changchun, China

**Keywords:** mung bean seeds, deep learning, MobileNet model, image classification, artificial intelligence

## Abstract

Mung bean seeds are very important in agricultural production and food processing, but due to their variety and similar appearance, traditional classification methods are challenging, to address this problem this study proposes a deep learning-based approach. In this study, based on the deep learning model MobileNetV2, a DMS block is proposed for mung bean seeds, and by introducing the ECA block and Mish activation function, a high-precision network model, i.e., HPMobileNet, is proposed, which is explored to be applied in the field of image recognition for the fast and accurate classification of different varieties of mung bean seeds. In this study, eight different varieties of mung bean seeds were collected and a total of 34,890 images were obtained by threshold segmentation and image enhancement techniques. HPMobileNet was used as the main network model, and by training and fine-tuning on a large-scale mung bean seed image dataset, efficient feature extraction classification and recognition capabilities were achieved. The experimental results show that HPMobileNet exhibits excellent performance in the mung bean seed grain classification task, with the accuracy improving from 87.40% to 94.01% on the test set, and compared with other classical network models, the results show that HPMobileNet achieves the best results. In addition, this study analyzes the impact of the learning rate dynamic adjustment strategy on the model and explores the potential for further optimization and application in the future. Therefore, this study provides a useful reference and empirical basis for the development of mung bean seed classification and smart agriculture technology.

## 1 Introduction

As a multi-nutrient-rich ingredient (Dahiya et al., 2015), mung bean has gained prominence in both traditional Chinese medicine theory and modern nutritional science (Ganesan et al., 2018). Analyzed from a nutritional perspective, mung beans are rich in unsaturated fatty acids and dietary fiber, which play a key role in regulating blood lipids, especially by lowering serum cholesterol and triglyceride levels, with potential benefits in preventing the occurrence and development of cardiovascular diseases (Ganesan et al., 2018). In dietary applications, the diverse cooking methods of mung bean, such as mung bean porridge, mung bean soup, and mung bean cake, not only enrich people’s dietary choices but also enhance the overall nutritional value through the complementary effects between ingredients, providing abundant options for a healthy diet (Liyanage et al., 2018).

The classification system of mung bean is essential for the accurate identification of its diversity, not only to facilitate consumers’ choice of appropriate mung bean varieties for consumption according to their personal preferences and health needs (Tarahi et al., 2024) but also to promote in-depth research in nutritional and food sciences. Specifically, the differences in nutrient composition and bioactive substances among mung bean varieties, such as protein composition, fiber content, vitamin types, and antioxidant capacity, have been clarified through the systematic classification, which provides a scientific basis for assessing the nutritional value and functional properties of mung bean varieties and provides strong support for the development of healthy dietary strategies for the public. In addition, classification is also important for agricultural practices. It helps farmers and growers understand the adaptive characteristics of mung bean varieties (Bellemare and Lim, 2018), including the optimal planting area, growth cycle, and cultivation management techniques so that they can precisely select seeds and optimize the planting structure according to the regional climate, soil conditions, and market demand, and realize the double enhancement of yield and economic benefits. At the same time, taking into account the differences in the sensory characteristics of mung bean varieties (such as taste, size, and shape), the classification also promotes the refinement of the food processing industry, so that enterprises can choose the best mung bean raw materials based on the product characteristics, to develop a more diversified, unique flavor mung bean products to meet the diversified market demand. At the market level, the classification of mung beans promotes the unification of market standards and the improvement of trading rules enhances market transparency and fairness, and provides consumers with clearer and more comparable purchasing options. For scientific research, the classification work has revealed the genetic diversity of mung bean (Zhao et al., 2020) and the intrinsic links between its varieties, laying a solid foundation for genetic breeding research, which indicates that new varieties of mung bean with higher quality, higher yield, and stronger resistance may be cultivated in the future, thus promoting the sustainable development of the mung bean industry (Acquah et al., 2021).

Traditional methods for seed purity identification include morphological examination (Kanwal et al., 2022), chemical identification (Yu et al., 2022), electrophoretic techniques (Nikolić et al., 2012), and spectroscopic techniques (Mehdizadeh et al., 2024). However, these methods are generally time-consuming, require specialized personnel and equipment, are often subject to the subjective experience of the tester, and are relatively costly. In addition, the identification process may damage the samples. Therefore, there is a need to develop a fast, accurate, non-destructive and inexpensive method for classifying and identifying mung bean seeds.

Classification of mung beans using computer vision technology and deep learning technology is of far-reaching significance in many aspects (Ma et al., 2023). Different mung bean varieties differ more or less in color, size, and shape, but there are also many similarities, which makes it challenging to classify mung bean seeds. Through computer vision technology, we can quickly and accurately identify the variety, color, area, size, and other characteristics of mung beans for automated classification and screening (Li et al., 2018). This cannot only reduce the error and labor intensity of manual classification but also improve the accuracy and efficiency of classification (Naik et al., 2024), further promoting the development of agricultural modernization and intelligence. Through computer vision technology, we can perform non-contact inspection of mung beans, avoiding the pollution and damage problems that may exist in traditional inspection methods (Feng et al., 2020). In addition, computer vision to classify mung bean seeds also helps to promote the development of related industries. For example, in the food processing industry, the classification and screening of mung beans by computer vision technology can provide better quality raw materials for the subsequent processing and improve the quality and taste of the products.

Chuanqi Xie (Xie and He, 2018) utilized visible and near-infrared hyperspectral imaging for the classification of mung bean varieties, and the original hyperspectral images of mung beans were collected at wavelengths of 380-1023 nm. An extreme learning machine (ELM) model was established to classify the mung bean species using the full-spectrum wavelength. The Modified-Gram-Schmidt (MGS) method was used to identify the effective wavelengths. Based on the selected wavelengths, ELM and linear discriminant analysis (LDA) models were developed. All models performed well, with correct classification rates (CCRs) ranging from 99.17%-99.58% in the training set and 99.17%-100% in the test set. Mulan Wu (Wu et al., 2023) compared two techniques (i.e., near-infrared (NIR) and Raman spectroscopy) for source identification and quantification of nutrients in mung beans. The predictive ability of orthogonal partial least squares discriminant analysis models for NIR and Raman spectroscopy were 94.3% and 92.9%, respectively, indicating that both NIR and Raman spectroscopy are capable of differentiating mung beans from different sources. Lei He (He et al., 2023) developed and optimized Random Forest (RF) and Support Vector Machine (SVM) models for distinguishing mung beans from different climate zones and growing zones. The SVM model outperformed the RF model in predicting both climate zones and growing zones of mung beans, with accuracies of 100% and 98.72%, respectively. Jian Li (Li et al., 2024) built a corn seed dataset containing a total of 5,877 images from six categories and proposed a corn seed recognition model based on the improved ResNet50 framework. The ResStage structure, Efficient Channel Attention (ECA) mechanism, Depth Separable (DS) convolution, and Swish-PReLU hybrid activation function were introduced to improve the model, respectively. The results show that the model achieves 91.23% accuracy in corn seed classification, which exceeds other related models. Compared with the original model, the model improved the accuracy by 7.07%, reduced the loss value by 0.19, and reduced the number of parameters by 40%. Yufei Ge (Ge et al., 2024) proposed a publicly accessible dataset for categorizing rice seeds for hyperspectral imaging systems. The dataset contains six categories with nearly 10,000 seeds in each category. Based on the proposed dataset, an Instance difficulty-weighted K Nearest Neighbors algorithm (IDKNN) is further proposed, and the effects of different regions of interest (ROIs) on the classification results are also explored, and compared with 10 representative algorithms, the IDKNN algorithm has the best performance, especially in the ROIs of the whole seeds. Chunguang Bi (Bi et al., 2022) combined deep learning with machine vision and utilized the foundations of Swin Transformer to improve corn seed recognition. The study focuses on feature attention and multi-scale feature fusion learning. The experimental results show that the proposed network model, MFSwin Transformer, has the highest classification accuracy compared to other models with an average precision, recall, and F1 value of 96.53%, 96.46%, and 96.47%, respectively, on the test set with a parameter memory of 12.83 M. The proposed network model, MFSwin Transformer, has the highest classification accuracy compared to other models. Lei Zhou (Zhou et al., 2020) proposed a convolutional neural network-based feature selector (CNN-FS) to screen out the depth-target related spectral channels and designed a convolutional neural network with attention (CNN-ATT) framework for wheat seed classification. CNN-ATT obtained the highest performance in the comparison experiments. CNN-ATT achieved 93.01% accuracy using the full spectrum and maintained its high accuracy (90.20%) by training on 60 channels of features obtained through the CNN-FS approach. Ziliang Huang (Huang et al., 2022) designed a complete soybean seed classification process following a segmentation-categorization procedure. Image segmentation is performed by a popular deep learning method, Mask R-CNN, while the classification phase is performed through a network called Soybean Network (SNet). SNet is a lightweight model based on convolutional networks, which contains the Mixed Feature Recalibration (MFR) module. The MFR module is designed to improve SNet’s ability to represent damaged features, making the model focus more on critical regions. Experimental results show that the SNet model proposed in this study can achieve 96.2% recognition accuracy using only 1.29M parameters. Chao Xia (Xia et al., 2019) collected 400 to 1000 nm hyperspectral images of 1632 maize seeds (17 varieties) for classifying seed varieties. Fourteen features, including one spectral feature and 13 imaging features (i.e., five first-order and eight S-order texture features), were extracted from the hyperspectral image data, and a multilinear discriminant analysis (MLDA) algorithm was proposed to select the optimal wavelengths and to transform and reduce the classification features in order to improve the speed of the acquisition and processing of hyperspectral images. Experimental results show that the combined features based on the MLDA wavelength selection algorithm have high classification accuracy at the same number of wavelengths (varying between 5-15 wavelengths). Perez Mukasa (Mukasa et al., 2022) used an RGB camera to capture watermelon seed ploidy images, DD-SIMCA and SVM quadratic classifiers for single class classification, and a multivariate machine learning approach to develop a watermelon ploidy seed discrimination model. One-class classification with the DD-SIMCA and the SVM-quadratic models yielded triploid discrimination accuracies of 69.5% and 84.3%, respectively. Andrea Loddo (Loddo et al., 2021) has proposed a model called SeedNet for seed classification and has utilized several state-of-the-art Convolutional Neural Networks to make the most adequate and exhaustive comparison of the considered scenarios. In detail, two analyzed datasets for seed classification, the first with an accuracy of 95.65% and the second with an accuracy of 97.47%, have been obtained with better results. Also, he investigated the problem of deep learning-based retrieval with satisfactory results. Mikel Barrio-Conde (Barrio-Conde et al., 2023) examined the ability of deep learning (DL) algorithms to classify sunflower seeds. Using a Nikon camera and an image acquisition system with controlled lighting, 6,000 seeds of six sunflower varieties were positioned and photographed. A CNN AlexNet model was used for variety classification, specifically categorizing 2 - 6 varieties. The classification model achieved a 100% accuracy value for two categories and 89.5% accuracy value for six categories, this result verifies that the deep learning algorithm has better classification results for sunflower seeds. Helong Yu (Yu et al., 2024) improved for ResNet50 and also improved the SE attention mechanism to obtain HResNet, which can effectively identify the origin of rice seeds. After a large number of comparisons and validations, HResNet obtained an accuracy of 95.13%, which is ahead of other comparative models and provides a reference for origin identification of other crops.

Based on the above studies, effective identification of seed or seed varieties is challenging due to similar appearance, genetic diversity, and growth environment. Therefore, the combination of neural networks and hyperspectral data has become a major tool for the effective identification of seed varieties. Although this identification method achieves good results, acquiring hyperspectral data is expensive and the processing involved is complex, making it difficult to realize large-scale applications. In response to the large number of studies on computer vision, most scholars are unable to simultaneously satisfy higher accuracy, lower number of parameters, and lower computational effort. To address these limitations, this study proposes a convolutional neural network model for mung bean seed recognition based on an image dataset of mung bean seeds, which is faster and more affordable relative to spectra by improving the lightweight network model MobileNetV2 (Sandler et al., 2018).

MobileNetV2 is chosen as the base model for the improved model mainly based on its comprehensive advantages. MobileNetV2 is a lightweight convolutional neural network designed for mobile and embedded vision applications, which incorporates innovative techniques such as depth convolution, inverted residual structure, and linear bottleneck layer to achieve efficient computational performance and excellent model performance. The main contributions and innovations of this paper are as follows.

1. In this study, a deep convolution module i.e. DMS block (DWConv-Mish-Sigmoid) is proposed for mung bean seeds and is introduced in the mid-stage of bottleneck to improve the feature extraction and network representation capabilities of the model. This enables it to capture and communicate image features more efficiently.
2. The ECA block was introduced in the later stages of the bottleneck (Wang et al., 2020). The attention mechanism strengthens the focus on the channel information and further enhances the ability of the model to capture more precise and detailed features, thus improving the efficiency of model recognition.
3. This study enhances the generalization ability and accuracy of the model in the prediction stage by introducing the Mish activation function (Misra, 2019), which enables it to better process the input data and make more accurate predictions.
4. At the same time, this study proposes a learning rate dynamic adjustment strategy for mung bean seeds, i.e., “95-Gradient”, so that the model can complete convergence and fitting while obtaining higher accuracy.

## 2 Materials and methods

### 2.1 Data acquisition and pre-processing

**Figure 1:**
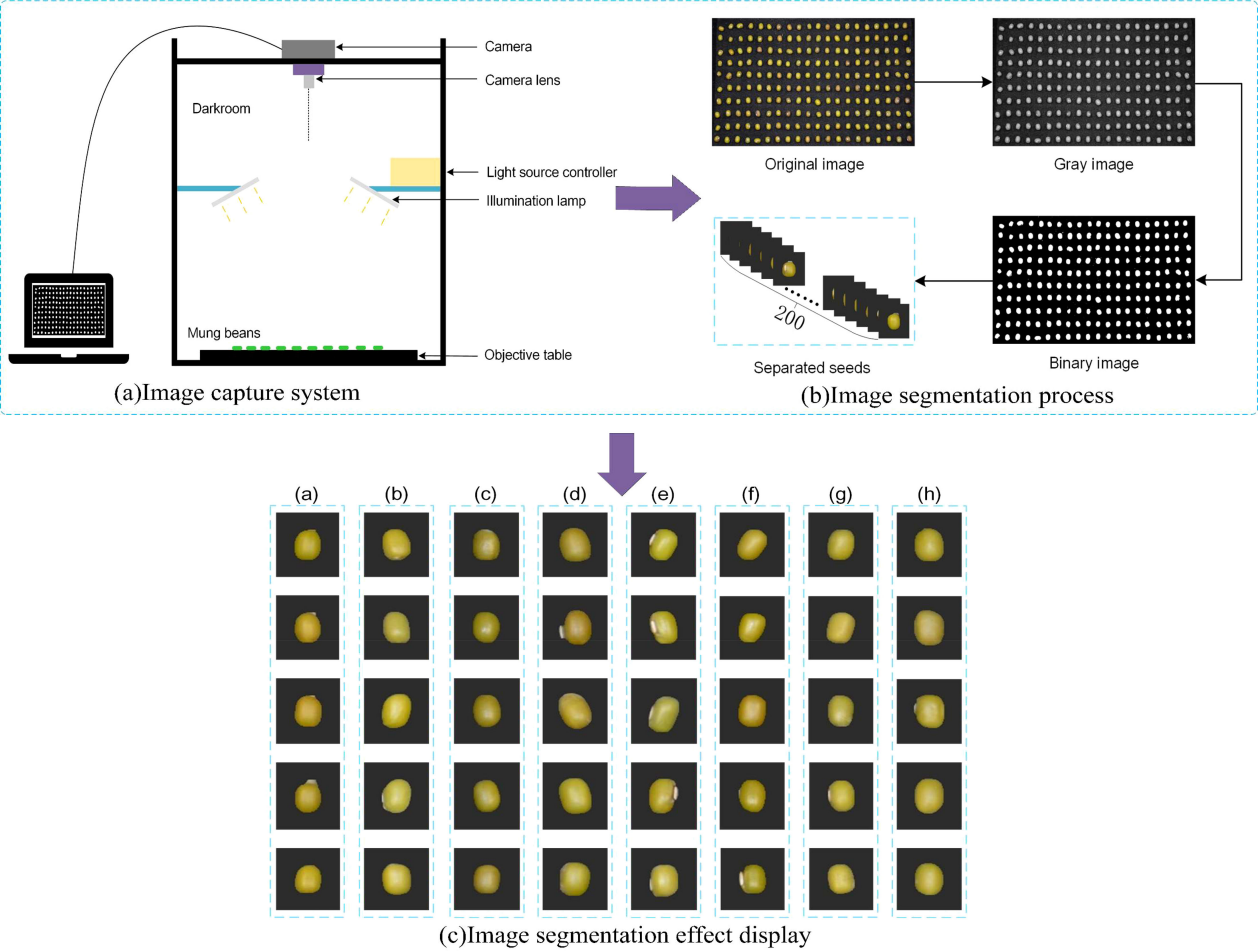
Image preprocessing process, **(A)** is image capture system, **(B)** is image segmentation process, **(C)** is image segmentation effect display. [The labels in **(C)** represent those in **Table 1**].

All samples in this experiment were provided by the Jilin Academy of Agricultural Sciences, China. A total of eight different varieties of mung beans were collected in this study, details of which are shown in **Table 1**. The imaging system as shown in **Figure 1A** consisted of a NikonD7100 camera with an 18-105mm lens, two lights controlled by a light source control system, a carrier table placed at the bottom to hold the mung bean seeds, and the camera was shot from vertical. During data collection, 200 mung bean seeds of the same variety were first randomly selected and arranged in a 10 × 20 grinding apparatus. Then, without seeds overlapping or adhering, they were inverted on a black absorbent cloth and their RGB images were acquired by the camera.

**Table 1:** Source of dataset varieties in this study.

Label	Variety Name	Code Name	Varietal Origin	Number of seeds
a	BaoLu.201323-3	BLC	Baoding Academy of Agricultural Sciences	1106
b	No. 07 GLu	GL07C	Hebei Academy of Agriculture and Forestry Sciences	930
c	No. 13 GLu	GL13C	Hebei Academy of Agriculture and Forestry Sciences	925
d	No. 05 JiLu	JL05C	Jilin Academy of Agricultural Sciences	965
e	No. 09 JiLu	JL09C	Jilin Academy of Agricultural Sciences	975
f	No. 10 JiLu	JL10C	Jilin Academy of Agricultural Sciences	690
g	No. 11 JiLu	JL11C	Jilin Academy of Agricultural Sciences	834
h	No. 06 JiLu	JL06C	Jilin Academy of Agricultural Sciences	975

The process of data preprocessing is shown in **Figure 1B**, using the threshold segmentation method. Firstly, based on taking the original image to obtain its grayscale image, and then selecting an appropriate threshold value to divide the pixels in the image into two categories (target and background), in this study, the threshold value is set to 0.4, and all the ones below 0.4 are set to 0 (black), and all the ones above 0.4 are set to 1 (white), which results in a binary image. The binary image is multiplied pixel by pixel with the original image so that only the pixels in the mask image with a value of 1 (representing the target region) are retained in the original image, while the pixels with a value of 0 (representing the background region) are set to black. Finally, the edges of the mung bean seeds are then extracted using the contour extraction algorithm based on the pixel distribution of the image with the background removed to extract the target region.

The effect of each variety of mung bean seeds after segmentation is shown in **Figure 1C**, where (a) to (h) respectively represent Labels in **Table 1**, which shows that different varieties of mung bean seeds have high similarity, which means that it poses a greater challenge to the classification performance of the model. In this experiment, useless images need to be eliminated after photographing the seeds, such as those that are blurred, damaged, or do not meet the experimental requirements at all, which may interfere with the training and evaluation process of the model and lead to the model learning erroneous or irrelevant features, and these images not only fail to provide valuable information for the model but also may degrade the model’s performance.

### 2.2 Data enhancement and dataset segmentation

Data enhancement is a very effective technique to generate more training samples by applying a series of transformations to the original image. Data enhancement not only increases the diversity of the dataset but also helps the model learn more different features, thus improving its generalization ability. Data enhancement enables the model to better adapt to various data variations (e.g., lighting, noise) in the real world by simulating these variations. This helps to improve the robustness of the model in complex environments or under different conditions, making it more reliable in real-world applications. For deep learning models, more training samples mean more learning opportunities, which helps to improve the accuracy and stability of the model. In this study, four data enhancement methods were selected, which are increasing brightness, adding noise, image mirroring, and image rotation.

**Figure 2:**
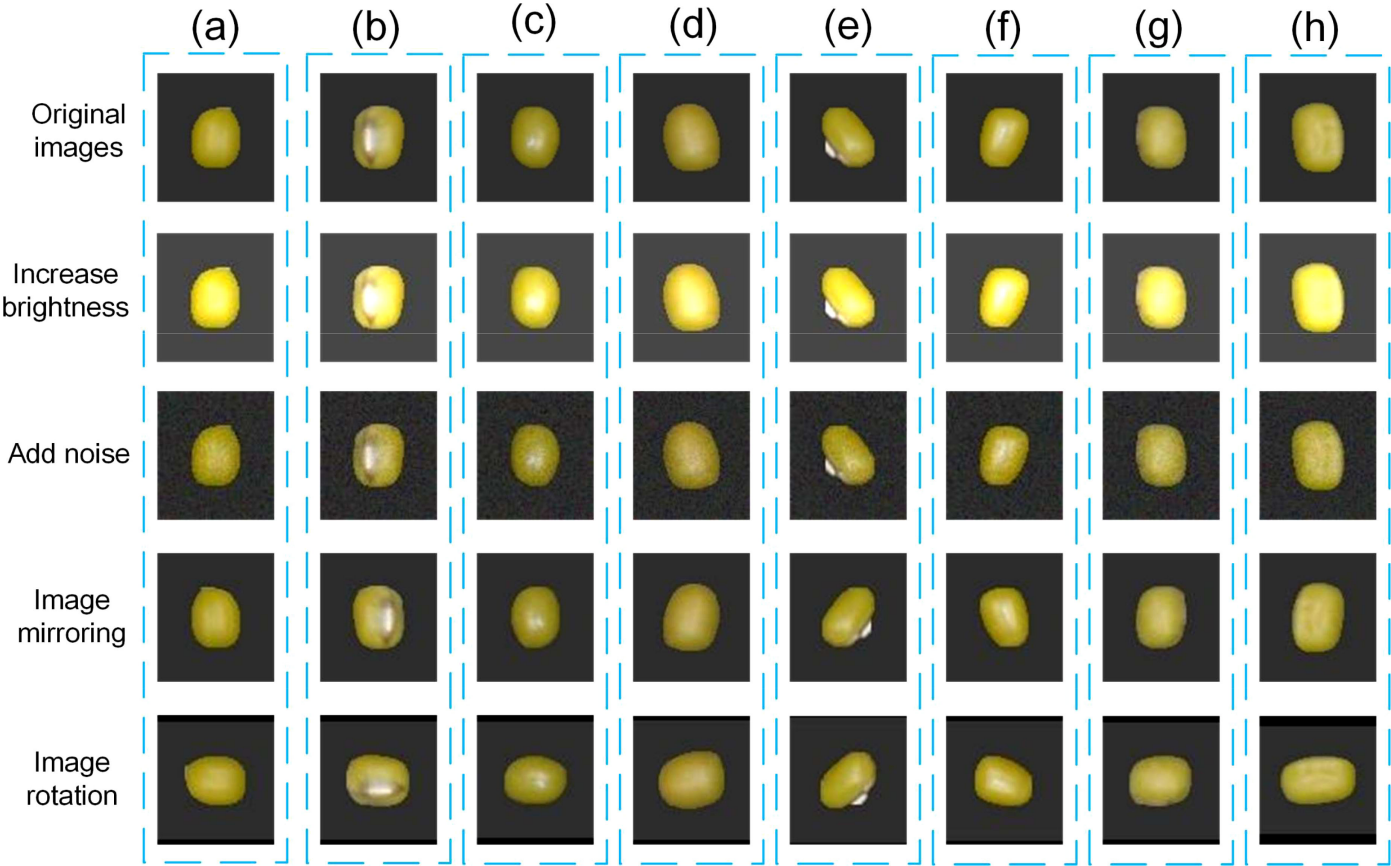
Demonstration of the effect of the data enhancement method. [Where **(A-H)** represent the labels in **Table 1**].

In this study the *ImageEnhance.Brightness* class from the PIL library was used to enhance the brightness of the image by setting the *brightness* variable to 2. This value represents the multiple of the enhancement, meaning that the brightness of the image was increased to two times the original brightness. The *np.random.normal* function is also used to generate Gaussian distributed random numbers with mean 1 and standard deviation 1.5, which are considered as noise and added to the original image. At the same time, image mirroring and rotating are also based on the functions in PIL, setting the appropriate mirroring direction and rotation angle to generate the corresponding image and save it to the corresponding folder. **Figure 2** shows the effect of each enhancement method, and these treatments tested the model’s ability to recognize mung beans under exposure conditions, its ability to deal with noise, and its ability to recognize mung beans at different angles, respectively.

After removing the useless images, data enhancement is performed and the enhanced dataset is randomly divided into a training set, validation set, and test set in the ratio of 8:1:1, the details of which are shown in **Table 2**. There is a significant imbalance in the number of samples of the eight mung beans in our dataset. An unbalanced dataset may lead to poor predictive performance of the model for a smaller number of samples. However, this study significantly expands the number of datasets through data enhancement, which can effectively compensate for this shortcoming.

**Table 2:** Data enhancement and segmentation details.

Label	Code Name	Original Image	Effective Image	After Enhancement	Training Set	Validation Set	Test Set
1	BLC	1106	1034	5170	4136	517	517
2	GL07C	930	881	4405	3524	440	441
3	GL13C	925	839	4195	3356	419	420
4	JL05C	965	860	4300	3440	430	430
5	JL09C	975	917	4585	3668	458	459
6	JL10C	690	685	3425	2740	342	343
7	JL11C	834	808	4040	3232	404	404
8	JL06C	975	954	4770	3816	477	477
/	Total	7400	6978	34890	27912	3487	3491

The label in the table represents the label of each category when the model is trained.

### 2.3 Model building

#### 2.3.1 Convolution in this study

**Figure 3:**
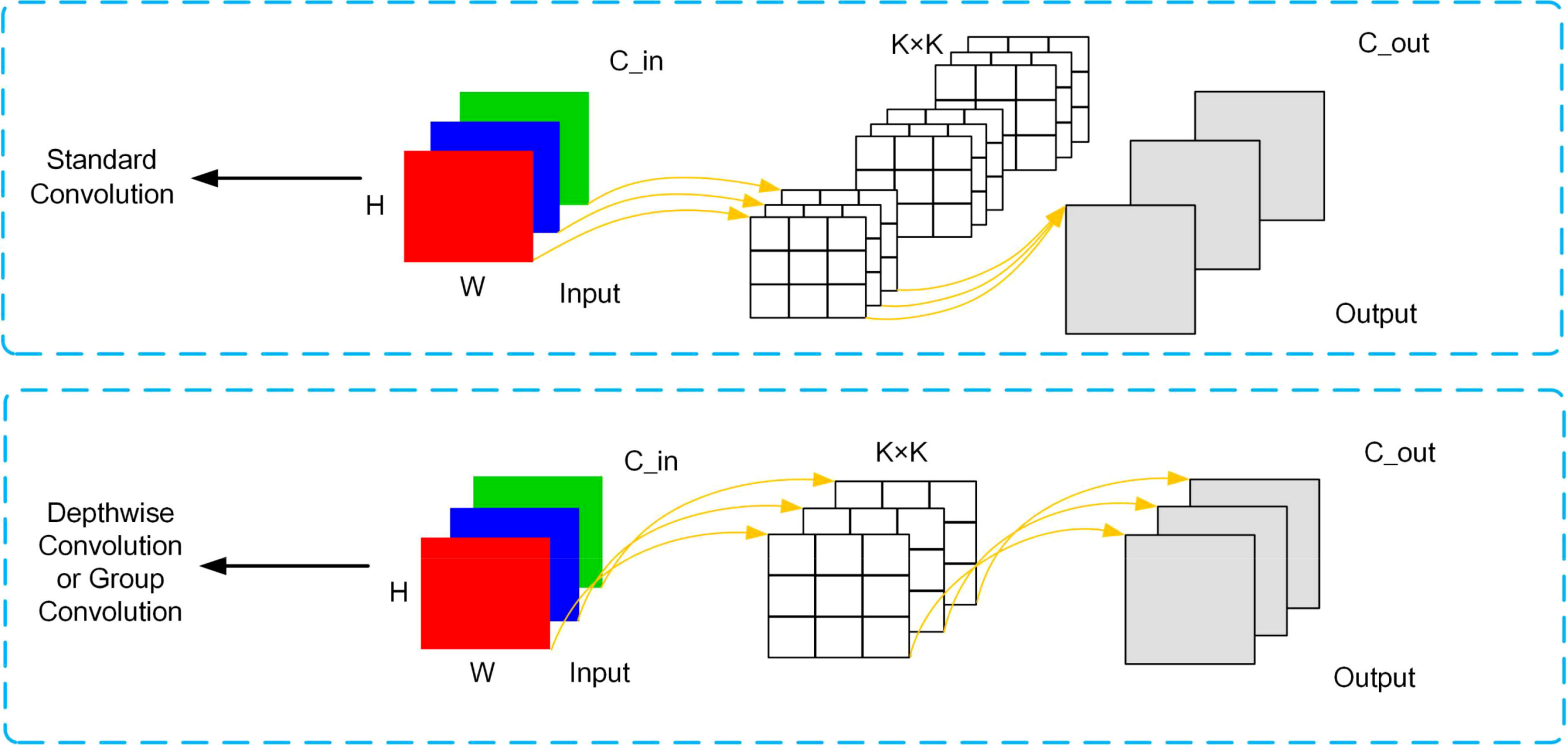
Schematic of Standard Convolution(Conv) and Depthwise Convolution/Group Convolution(DWConv/GConv).

As shown in **Figure 3**, where the number of input and output channels are *C_in* and *C_out* respectively, *K×K* is the kernel size of the convolution, and the size of the image is *H×W*. DWConv (Depthwise Convolution) processes each channel of the input feature map separately, which reduces the number of convolution kernels compared to Conv (Standard Convolution). This significantly reduces the number of parameters and reduces the model complexity. DWConv performs only a single-channel convolution operation for each channel, so the computational effort is relatively small, compared to normal convolution which requires all channels to be processed at the same time, and the computational effort is much larger. Due to the reduction in the number of parameters and computation, using DWConv can improve the efficiency of the model while maintaining reasonable performance, which is especially important in applications where resources are limited or real-time response is required, such as mobile devices or edge computing scenarios. Their parameters and FLOPs (Floating Point Operations) computational details are shown in **Table 3**, where G is the size of the group. The main difference between DWConv and GConv is that when *G* is greater than 1 and less than *C_in*, then it is a group convolution, when *C_in* is equal to *G*, it is a deep convolution, and when *G* is equal to 1 it is a standard convolution.

**Table 3:** Conv and DWConv/GConv comparison of parameters and FLOPs.

Type of Convolution	Parameters	FLOPs
Conv	$K^{2}\times C\_in\times C\_out$	$K^{2}\times C\_in\times H\times W\times C\_out$
GConv	$K^{2}\times \frac{C\_in}{G}\times C\_out$	$K^{2}\times \frac{C\_in}{G}\times H\times W\times C\_out$
DWConv	$K^{2}\times \frac{C\_in}{C\_in}\times C\_out$	$K^{2}\times \frac{C\_in}{C\_in}\times H\times W\times C\_out$

#### 2.3.2 Mish activation function and ReLU6 activation function

**Figure 4:**
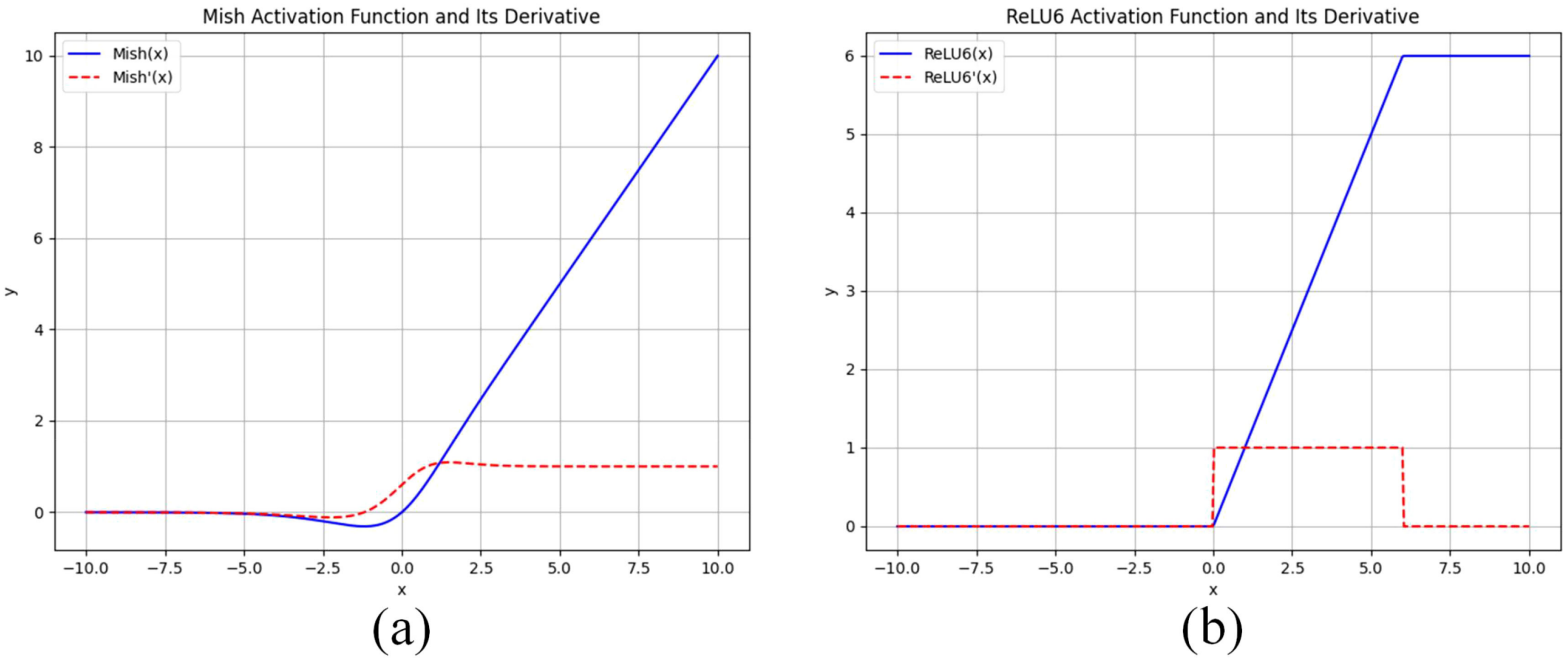
**(A)** Shows the graph of Mish’s activation function and its derivatives, and **(B)** Shows the graph of ReLU6’s activation function and its derivatives.

A comparison of the graphs of the Mish and ReLU6 activation functions and the graphs of their derivatives is shown in **Figure 4**, where the Mish activation function is a smooth nonlinear function, in contrast to ReLU6, which is segmented linear. The smoothness implies that Mish is differentiable throughout the entire domain of definition, which helps the optimization algorithm to be more stable and efficient during the training process. In addition, the Mish function is non-monotonic, which makes it potentially more expressive than monotonic ReLU6 when dealing with complex nonlinear data. The ReLU6 activation function has a gradient of 0 when the input is negative, which may lead to gradient vanishing problems, especially in deep neural networks. The formula of its activation function is shown in **Table 4**.

**Table 4:** Details of the activation function formula used in this study.

Activation Functions	Formulas
ReLU6	$f\left(x\right)=\{\begin{array}{c} 0,\;\;x\leq 0\\ x,0<x\leq 6\\ 6,\;\;x>6 \end{array}$
Mish	f(x)=x×tanh (softplus(x))
Tanh	$f\left(x\right)=\frac{e^{x}-e^{-x}}{e^{x}+e^{-x}}$
Softplus	$f\left(x\right)=\text{ln}\;\left(1+e^{x}\right)$
Sigmoid	$f\left(x\right)=\frac{1}{1+e^{-x}}$

#### 2.3.3 Loss function (Cross Entropy Loss)

The loss function used in this study is Cross Entropy Loss, Cross Entropy Loss is robust to the probability distribution predicted by the model. Even if the model has a small deviation in the predicted probability of some categories, it will not affect the overall loss too much. This makes the model more stable during training and less susceptible to noise or outliers. The binary classification Cross Entropy Loss is shown below:


(1) $$\begin{array}{c} L=\;-\left[y\log p+\left(1-y\right)\log\left(1-p\right)\right] \end{array}$$

Where y denotes the sample label and p denotes the probability that the corresponding sample label is predicted to be positive. In the multiclassification task, each sample may have more than one possible category, and the model output is the probability distribution of each sample belonging to each category, Cross Entropy Loss can measure the distance between the probability distribution of the model output and the true labels, to guide the model optimization. The multicategory Cross Entropy Loss formula is shown below:


(2) $$\begin{array}{c} L=\;-\sum_{c=1}^{M}y_{c}\log p_{c} \end{array}$$

where $p_{c}\;$ denotes the probability that the label is predicted to be c.

#### 2.3.4 DMS block and bottleneck

**Figure 5:**
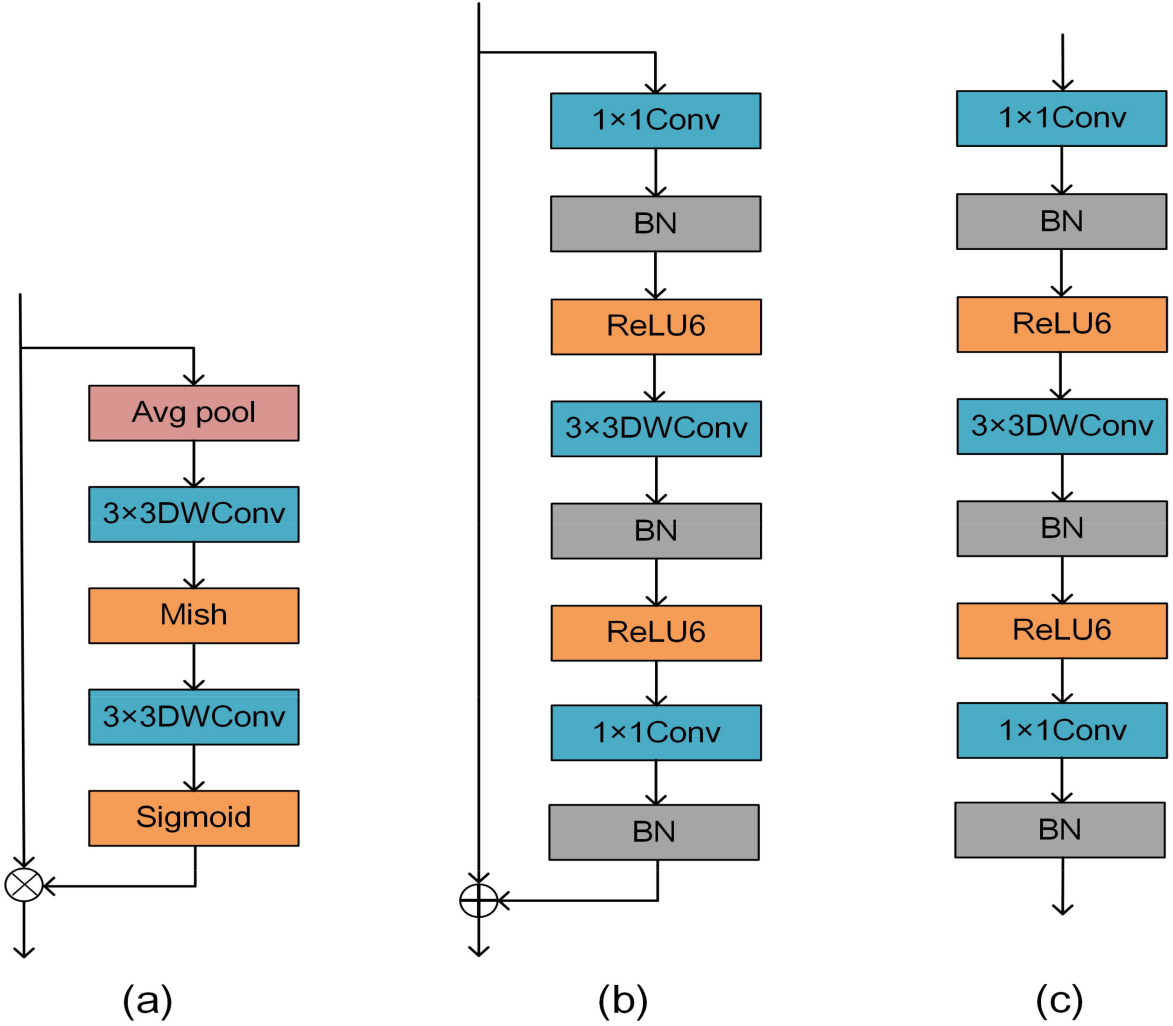
**(A)** Shows the structure of the DMS block, **(B)** shows the bottleneck in MobileNetV2 with stride=1, and **(C)** shows the bottleneck with stride=2.

In this study, a depthwise convolution block i.e. DMS block (DWConv-Mish-Sigmoid) is proposed for mung bean seeds as shown in **Figure 5A**. Firstly, a down-sampling operation is performed on the input data through the pooling layer to reduce the spatial size of each feature map, which reduces the computational effort of the subsequent layers and thus reduces the complexity of the overall block. Immediately after that, two 3×3 DWConv to extract features from the input data, and at the end residual joins are introduced. Two different types of activation functions, Mish and Sigmoid, are used in the DMS block, to introduce different nonlinear properties, which help the network to learn more complex and abstract feature representations.

**Figure 5B** demonstrates the bottleneck in MobileNetV2 with stride=1, and **Figure 5C** demonstrates the bottleneck with stride=2. The bottleneck structure significantly enlarges the number of input channels by introducing the 1x1Conv, followed by the 3x3DWConv, and finally by the 1x1Conv to narrow the number of channels. This bottleneck design significantly reduces the computational complexity of each layer while maintaining a high feature representation and feature extraction capability. This structure also allows the network to better capture and represent abstract features in the image, thus improving the model’s performance in the mung bean seed recognition task. The introduction of the bottleneck structure allows the design of a deeper-level network without adding too many parameters and computational overhead. This flexibility allows MobileNetV2 to be extended and adapted to the task of mung bean seed classification and recognition while maintaining the efficiency and performance of the model.

#### 2.3.5 ECA (Efficient Channel Attention) block

**Figure 6:**
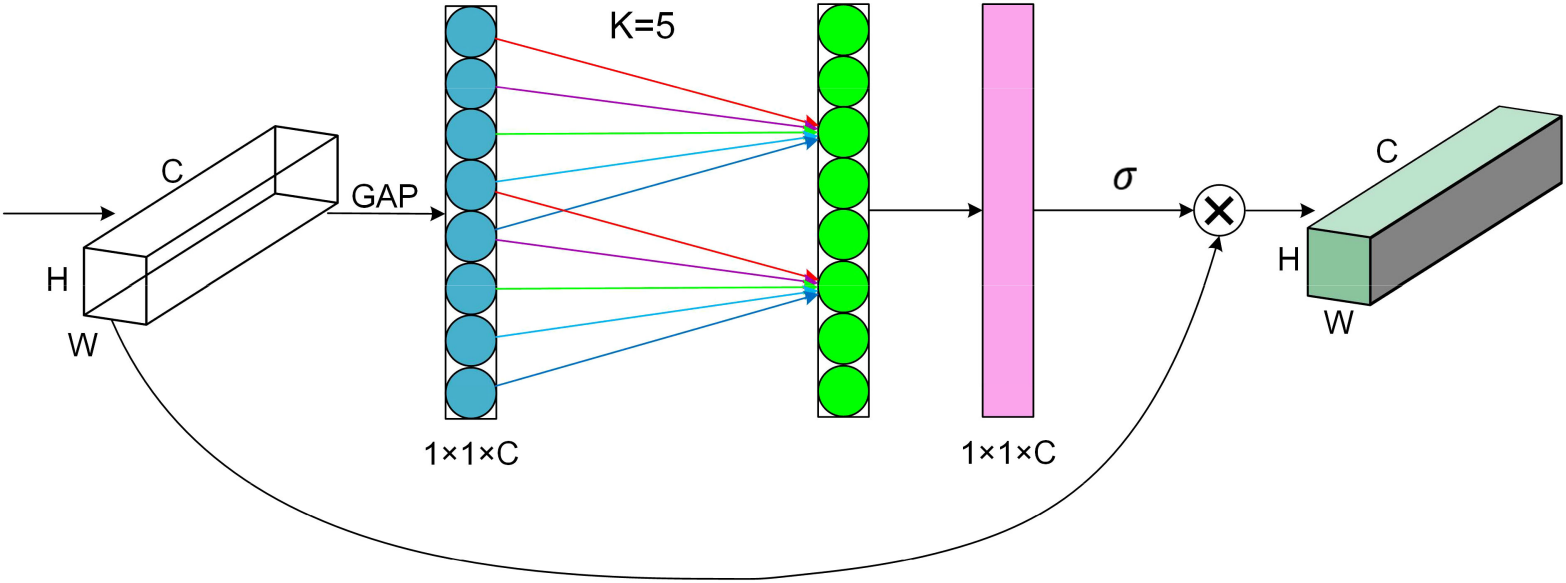
Structure of the efficient channel attention block.

The attention mechanism plays a crucial role in deep learning to efficiently and accurately filter out valuable information from massive data, which is very beneficial for various image processing tasks. Therefore, in this study, we introduce an attention mechanism called ECA (Efficient Channel Attention). The ECA block adaptively adjusts the weights of the channel features so that the network can better focus on the essential features. Most mung bean seeds have similar shapes and textures, which makes it difficult to extract detailed features from the network. The ECA block helps to improve feature differentiation and suppresses unimportant features, thus reducing the risk of overfitting. Ultimately, the feature representation is enhanced and the generalization ability of the model is improved without significantly increasing the computational cost. The structure of the ECA block is shown in **Figure 6**.

The ECA block first performs global average pooling of input feature mappings of size H × W × C to obtain feature information during forward propagation. Then, new weights *ω* are generated by one-dimensional convolution of size *K* and Sigmoid activation function to complete the inter-channel information interaction, as shown in Equation 3.


(3) $$\begin{array}{c} \omega =\;\sigma \left(C1D_{k}\left(y\right)\right) \end{array}$$

where $C1D_{k}$ Denotes a one-dimensional convolution with kernel size *k* and *σ* denotes the Sigmoid activation function. As shown in Equation 4, the number of channels *C* is proportional to the one-dimensional convolution with the kernel of *k*.


(4) $$\begin{array}{c} C=\;2^{\left(\gamma *k-b\right)} \end{array}$$

Therefore, we can obtain the final kernel size *k* as shown in Equation 5.


(5) $$\begin{array}{c} k={\left|\frac{\log_{2}\left(C\right)}{\gamma }+\frac{b}{\gamma }\right|}_{odd} \end{array}$$

Where *t* is the nearest odd number to ${\left|t\right|}_{odd}$ , γ is 2, and *b* is 1.

#### 2.3.6 MobileNetV2 and HPMobileNet

**Figure 7:**
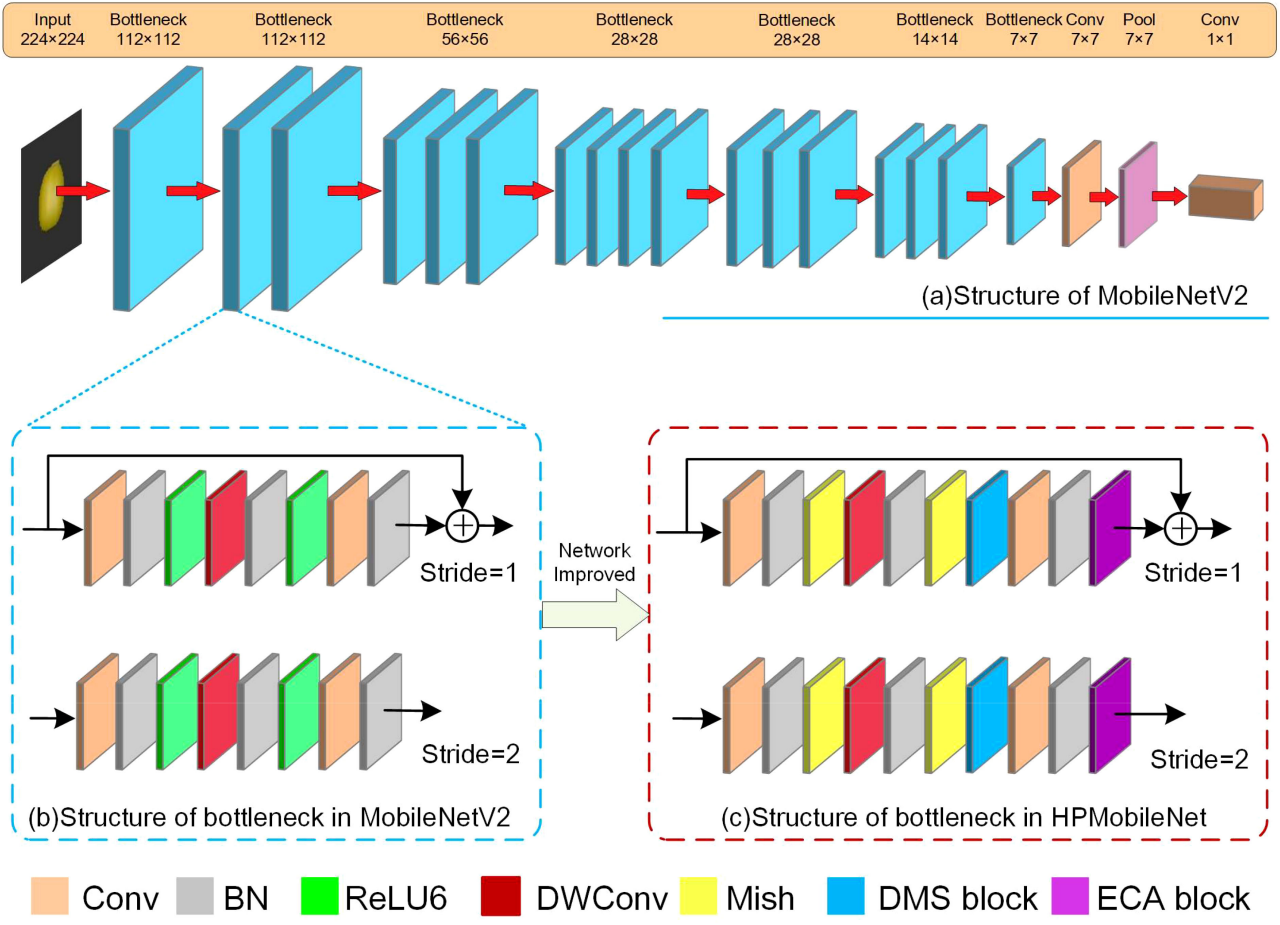
The improved model, **(A)** is the structure of MobileNetV2, **(B)** is the structure of bottleneck in MobileNetV2, **(C)** is the structure of bottleneck in HPMobileNet.

The improvement process of the model is shown in **Figure 7**, where **Figure 7A** shows the original structure of the MobileNetV2 model, **Figure 7B** shows the structure of the bottleneck in the MobileNetV2 model, and **Figure 7C** shows the bottleneck structure of the improved model, HPMobileNet, where BN stands for the batch normalization layer, which replaces the ReLU6 activation function is replaced by the Mish activation function. In bottleneck, the first convolution has the role of upscaling, using the second convolution (DWConv) for feature extraction and then downscaling by the third convolution, the DMS block is put behind the second batch normalization to extract more features in conjunction with DWConv. The ECA block is placed after the last batch normalization in the bottleneck to make it gather features among the previously extracted feature information, which helps the model to focus on the most important parts of the input data, thus improving the performance of the model.

The parameter settings within the HPMobileNet model are shown in **Table 5**, which uses a similar parameter structure to MobileNetV2, where *t* is the expansion factor, *c* is the output feature matrix depth channel, *n* is the number of repetitions of the bottleneck, bottleneck here refers to the inverted residual structure, and *s* is the step size.

**Table 5:** Details of parameter settings within the HPMobileNet model.

Input	Operator	t	c	n	s
$224^{2}$ × 3	conv2d	–	32	1	2
$112^{2}$ × 32	bottleneck	1	16	1	1
$112^{2}$ × 16	bottleneck	6	24	2	2
$56^{2}$ × 24	bottleneck	6	32	3	2
$28^{2}$ × 32	bottleneck	6	64	4	2
$14^{2}$ × 64	bottleneck	6	96	3	1
$14^{2}$ × 96	bottleneck	6	160	3	2
$7^{2}$ × 160	bottleneck	6	320	1	1
$7^{2}$ × 320	conv2d 1×1	–	1280	1	1
$7^{2}$ × 1280	avg pool × 7	–	–	1	–
1 × 1 × 1280	conv2d 1 × 1	–	k	–	–

### 2.4 Model evaluation indicators

In the field of machine learning, confusion matrices are often used to compare the results of model classification in supervised learning. Each column of the matrix represents the predicted class and each row represents the actual class. Take the binary classification problem as an example, define that the actual result is positive and the predicted result is positive, denoted as TP; if the actual result is negative, the predicted result is positive, denoted as FP; if the actual result is positive, the predicted result is negative, denoted as FN; and if the actual result is negative, the predicted result is negative, denoted as TN. The specific structure of the confusion matrix is shown in **Supplementary Table S1**.

Accuracy (Acc), precision (P), recall (R), and F1 score (F1) can be computed from the data in the confusion matrix and used as evaluation metrics for assessing the classification performance of the model. The formulas and short descriptions of these evaluation metrics are shown in **Table 6**.

**Table 6:** Calculation formulas of each indicator.

Evaluation indicators	Formulas	Brief Description
Accuracy (Acc)	$Acc=\;\frac{TP+TN}{TP+FP+FN+TN}$	The ratio of the number of correctly predicted positive and negative samples to the total number of samples.
Precision (P)	$P=\;\frac{TP}{TP+FP}$	The ratio of the number of correctly predicted positive samples to the total number of samples predicted to be positive.
Recall (R)	$R=\;\frac{TP}{TP+FN}$	The ratio of the number of correctly identified positive samples to the total number of actual positive samples.
F1-score (F1)	$F1=\;\frac{2TP}{2TP+FP+FN}$	The reconciled mean of precision and recall.

### 2.5 Hyperparameter information for model training

This study provides information on the specific experimental parameters used in training the new network model proposed in this paper. We set the input size of the dataset to 224 × 224, the number of training rounds to 100, the optimizer to use SGD, and the batch size to 64. details are shown in **Supplementary Table S2**.

If the learning rate is set too high or too low, it can have a big impact on the model’s learning process. If the learning rate is set too high, the advantage is that the model may update the weights faster and thus explore the space of possible solutions faster. But then, the disadvantage is also obvious that the model may miss the optimal solution because the step size is too large, leading to oscillations or even divergence during the training process, making it difficult to converge to a stable solution. On the contrary, if the learning rate is set too low, although the model can converge more stably, the speed of convergence may be very slow, the advantage is that the model may adjust the weights more finely to get a more accurate solution, but the disadvantage is also obvious, the training process will become very time-consuming, and the model may be more likely to fall into the local optimal solution, and cannot find the global optimal solution. Therefore, it is very important to set the appropriate learning rate, so we choose the gradient decay strategy to dynamically adjust the learning rate during the training process to achieve better training results, the initial learning rate is set to 0.01, and the learning rate is set to decay every 2 rounds, and the decay rate is set to 0.95, which is named as “95-Gradient “.

### 2.6 Experimental environment configuration

This experiment was deployed on a computer with an Intel(R) Xeon(R) Gold 6246R CPU (3.4GHZ) and NVIDIA Quadro RTX 8000 GPU (48GB) with Windows 10 operating system, with software configuration installed as Anaconda 3-2021.11-windows version with PyCharm compiler and given PyTorch 1.2.1 with built-in Python 3.8.3 programming language, and all the algorithms are run in the same environment as shown in **Supplementary Table S3**.

## 3 Results

### 3.1 Removal of unwanted images and data enhancement from the model

MobileNetV2 is used as the base model to explore the classification results of the original image dataset, the dataset after removing the useless images, and the dataset after data enhancement, as shown in **Table 7**. The results show that the accuracy of the original image is only 76.02%, and the loss reaches 0.685. After removing the useless images, the accuracy is slightly improved to 77.81%, and the loss is also slightly reduced to 0.632, which indicates that the blurred, damaged, or completely non-compliant images do interfere with the training and evaluation process of the model and lead to the model to learn the wrong or irrelevant features. These images not only fail to provide valuable information for the model but also reduce the performance of the model. After performing data enhancement, the accuracy rate was substantially increased to 86.92% and the loss was reduced to 0.354. This indicates that the use of data enhancement can effectively expand the diversity of data and enhance the generalization ability of the model. Therefore, the data-enhanced mung bean samples were used for the next experiment.

**Table 7:** Comparison of results between original images, effective images, and after data enhancement.

Data Set	Data Volume	Acc (%)	Loss
Original Images	7400	76.02	0.685
Effective Images	6978	77.81	0.632
After Data Enhancement	34890	86.92	0.354

### 3.2 Comparison of experimental results

#### 3.2.1 Learning rate dynamic adjustment strategy vs. selected learning rates

**Figure 8:**
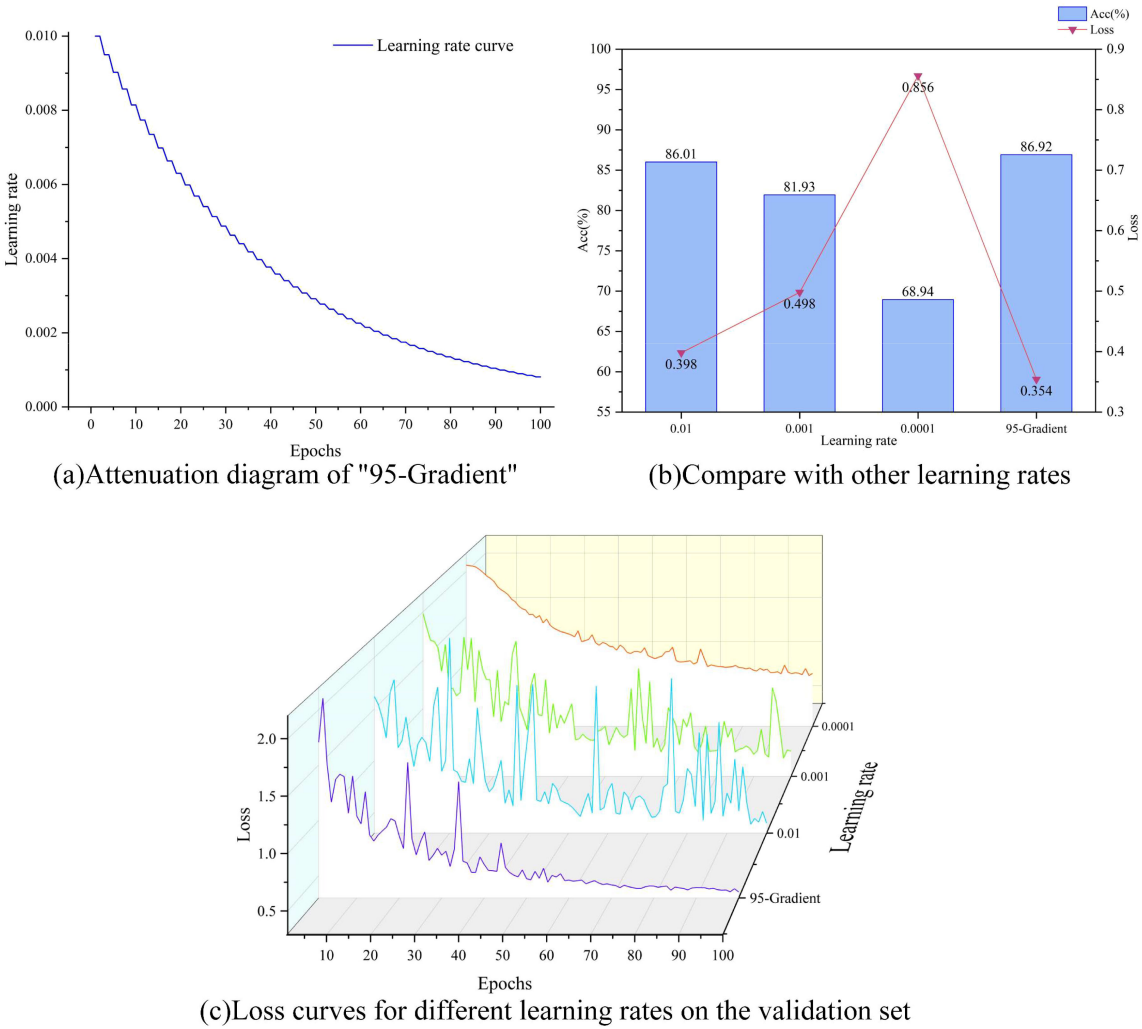
"95-Gradient " effect contrast, **(A)** shows the attenuation diagram of "95-Gradient", **(B)** Comparison of accuracy and loss between "95-Gradient" and several other commonly used learning rates, and **(C)** is loss curves for different learning rates on the validation set.

The attenuation effect of the learning rate dynamic adjustment strategy “95-Gradient “ is shown in **Figure 8A**. The learning rate adjustment strategy of decaying every two rounds helps the model to converge gradually during the training process. At the beginning of training, a larger learning rate can help the model approach the optimal solution quickly. As the training progresses, the gradual decrease of the learning rate allows the model to search near the optimal solution with a smaller step size, which improves the accuracy and stability of convergence. Taking MobileNetV2 as the base model, as shown in **Figure 8B**, the model is first compared with three representative learning rates, and the results show that “95-Gradient” obtains the highest accuracy and the lowest loss, which are 86.92% and 0.354 respectively. Meanwhile, from **Figure 8C**, it can be seen that when the learning rate is equal to 0.01 and 0.001, the accuracy of the model fluctuates continuously during the training process, which may be because the optimal solution is missed because of the too-large step size, resulting in continuous oscillation or even dispersion during the training process, and it is difficult to converge to a stable solution. When the learning rate is equal to 0.0001, the model cannot converge to the global optimal solution because the step size is too small, resulting in the model falling into the local optimal solution during the training process, which makes the accuracy lower. This problem can be effectively solved by adding the “95-Gradient”, as the training progresses, the learning rate gradually decreases, and the model can reduce the step size when approaching the optimal solution to avoid excessive fluctuations. As a result, the convergence curve will become smoother in the later stage and gradually converge to the optimal solution.

This result shows that the learning rate dynamic adjustment strategy proposed in this study not only improves the convergence speed of the model but also enhances the stability of the model, which enables the model to achieve satisfactory performance in fewer iterations. However, large fluctuations also occur in the early stage of model training, and this problem is solved by changing the structure of the model. It can be concluded that the “95-Gradient” plays a crucial role in model training.

#### 3.2.2 Comparison results of different activation functions

**Figure 9:**
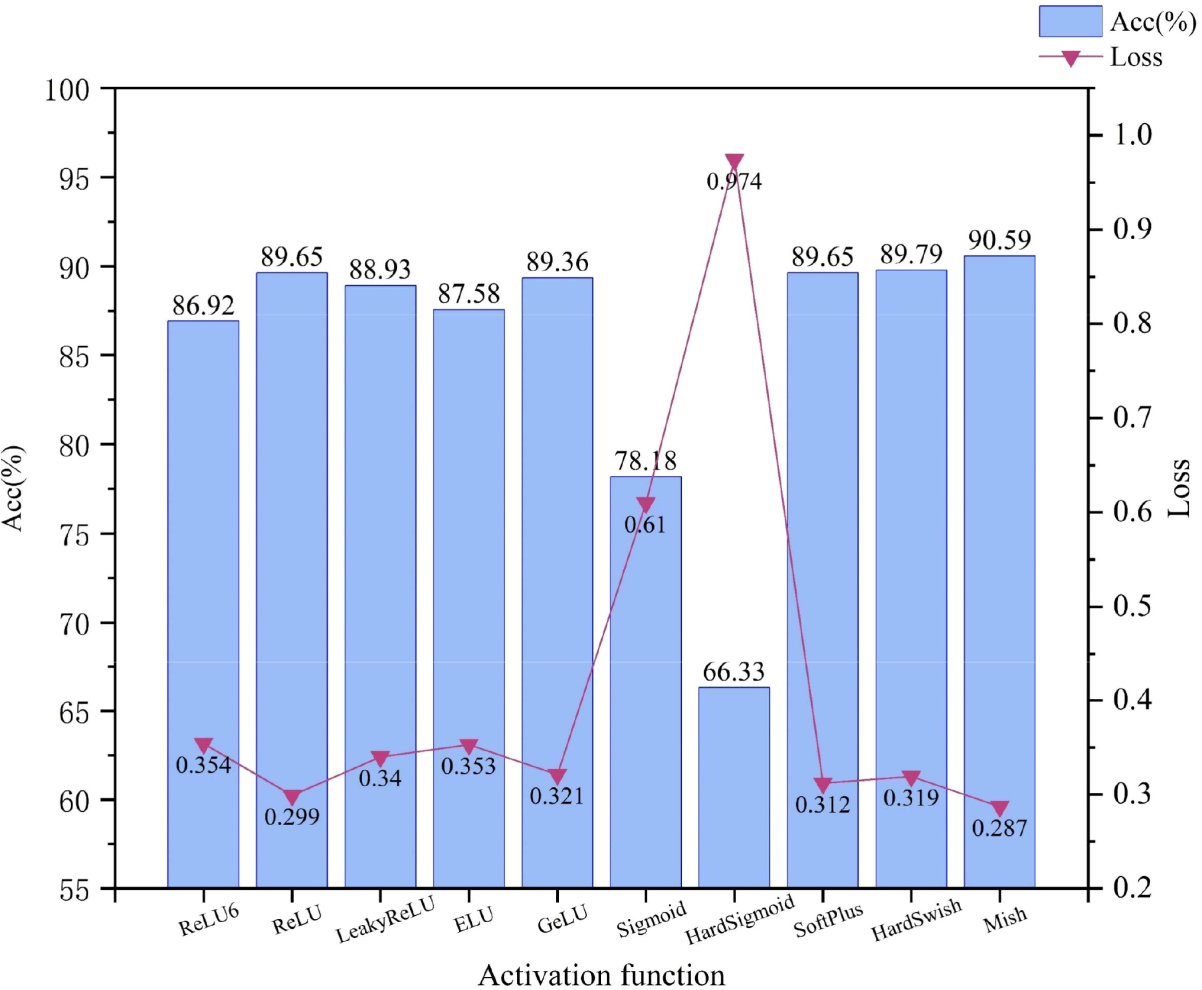
Comparative accuracy and loss results for different activation functions.

MobileNetV2 is used as the base model and compared with other common activation functions. **Figure 9** shows that the Mish activation function achieves the highest accuracy while achieving the lowest loss, with an improvement of 3.67% in accuracy and a reduction of 0.067 in loss, relative to the activation function ReLU6 used in the original model. This result suggests that the Mish activation function enables the neural network model to learn and represent more complex functional relationships by introducing nonlinear elements, thus enhancing the expressive and fitting capabilities of the model to maximize the differences between categories, such as the color, shape, and texture of mung bean seeds, enabling the extraction of challenging and detailed features. In this study, the advantage of the Mish activation function is fully utilized to obtain better generalization performance and recognition results of the model, which significantly improves the model’s classification performance for mung bean seeds.

#### 3.2.3 Comparative results of different attention mechanisms

**Figure 10:**
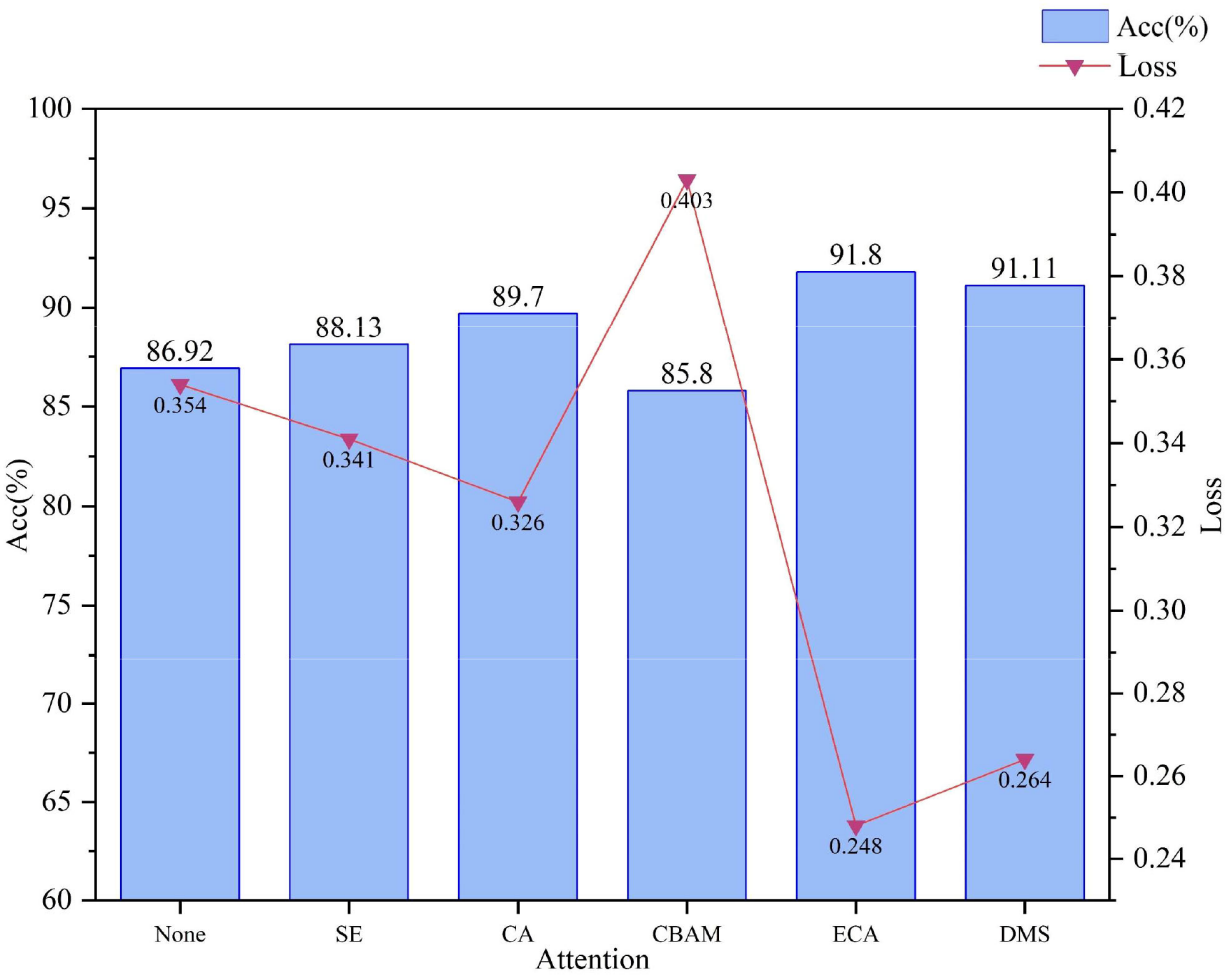
Comparative accuracy and loss results for different attention blocks.

Using MobileNetV2 as the base model, the ECA block and DMS block are compared with several other common attention mechanisms, respectively, and they are put into the same position as the ECA block to compare their performances respectively, and the results are shown in **Figure 10**, the ECA block and DMS block achieved the highest accuracy rate, 91.8%, and 91.11% while obtaining the lowest loss of 0.248 and 0.264, respectively. This result suggests that the ECA block and DMS block focus more on enhancing the network’s ability to model inter-channel dependencies by considering the importance of each channel globally. In a lightweight network such as MobileNetV2, the ECA block and DMS block can maximize the model’s ability in complex feature extraction and improve the network’s focus on key features, which further improves the model’s performance. The ECA attention mechanism utilizes a one-dimensional convolution to capture local cross-channel interactions, which is designed to take into account the characteristics of each channel while also being able to extract inter-channel dependencies. By learning the importance of each channel relative to the others, the ECA block can dynamically adjust the intensity of the channel’s response, thereby enhancing the model’s feature representation and hence its accuracy.

#### 3.2.4 Comparison results with classical network models on the test set

To validate the effectiveness and sophistication of the network model HPMobileNet proposed in this study, this study firstly uses the model accuracy, model parameters, model floating point operations per second (FLOPs), and the weight size of the model as the evaluation metrics of the model performance. We compare the new network model with eight classical network models (ResNet50, ResNeXt50, Res2Net50, MobileNetV3_S, GhostNet, RepVggNet_B0, ConvNext_T, and FasterNet_T0) to evaluate their performance. The details are shown in **Table 8**.

**Table 8:** Comparative results of different models.

Model	Acc (%)	Params (M)	FLOPs (G)	Weight Size (MB)
ResNet50 (He et al., 2016)	87.71	23.524	4.132	179
ResNeXt50 (Xie et al., 2017)	89.00	22.996	4.286	175
Res2Net50 (Gao et al., 2019)	87.34	14.327	2.394	109
MobileNetV3_S (Howard et al., 2019)	89.20	1.528	0.061	11.8
GhostNet (Han et al., 2020)	87.48	3.912	0.155	30.1
RepVggNet_B0 (Ding et al., 2021)	89.46	14.547	3.428	111
ConvNeXt_T (Liu et al., 2022)	65.63	27.805	4.455	212
FasterNet_T0 (Chen et al., 2023)	85.91	2.635	0.339	20.2
**HPMobileNet(Our)**	**94.01**	**2.370**	**0.329**	**18.4**

The bold values represent the results of the algorithm HPMobileNet proposed in this study.

The results show that the accuracy of HPMobileNet reaches 94.01%, which is ahead of other network models, with only 2.370M parameters, which is second only to MobileNetV3_S’s 1.528M, and only 0.329G FLOPs, which is second only to MobileNetV3_S’s 0.061G and GhostNet’s 0.155G, and the size of the weights generated by the network model is only 18.4MB, which is second only to MobileNetV3_S’s 11.8MB. Overall, the comprehensive ability of the network model proposed in this study achieves the best results, with both high accuracy and low time and space complexity, and its overall strength is ahead of other classical network models.

To compare the performance of model recognition more intuitively, this study visualizes and analyzes the comparison models, as shown in **Supplementary Figure S1**, which shows more clearly that HPMobileNet obtains the highest accuracy rate and is ahead of other models.

**Figure 11:**
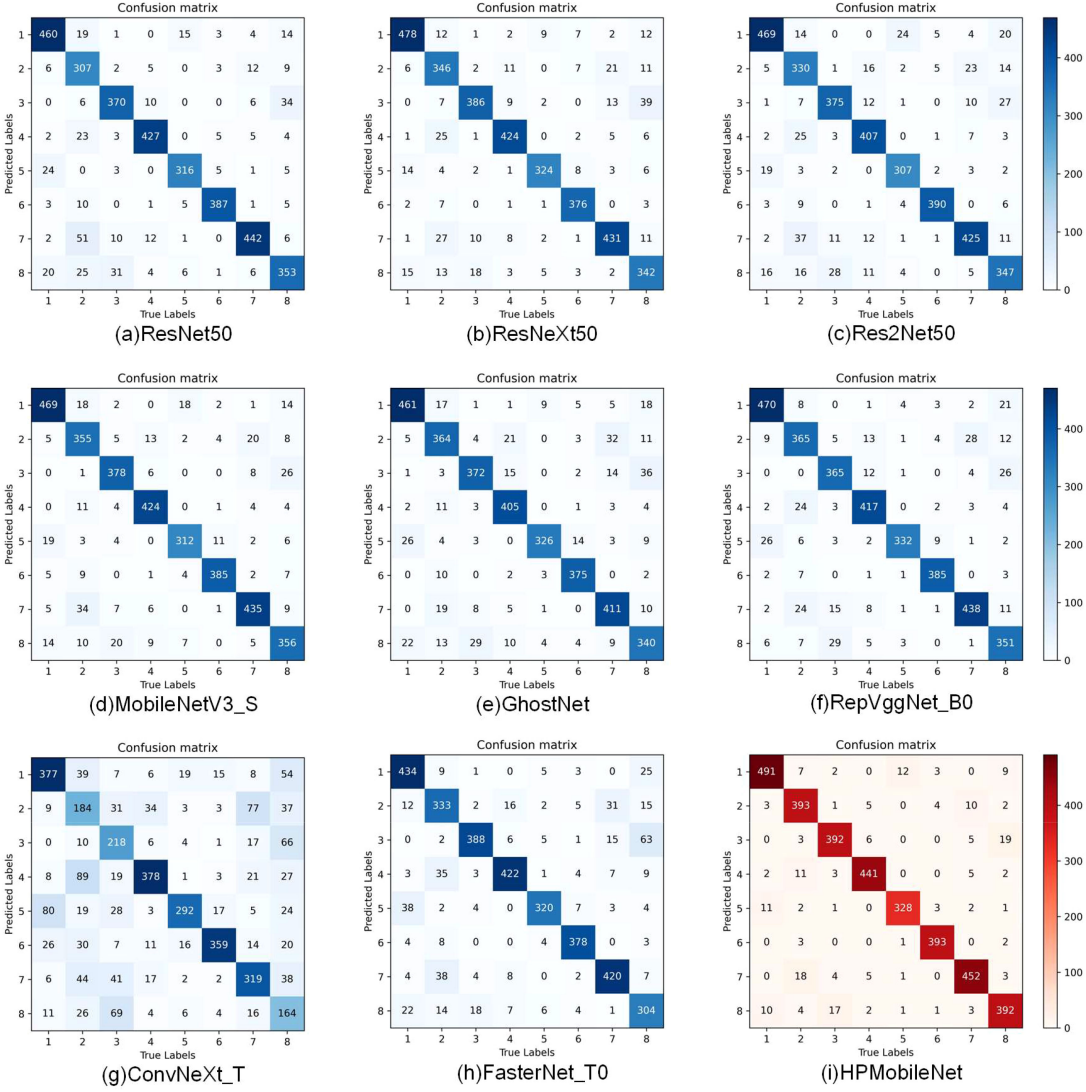
Comparison of confusion matrix for different models, **(A)** is the confusion matrix of ResNet50, **(B)** is the confusion matrix of ResNeXt50, **(C)** is the confusion matrix of Res2Net50, **(D)** is the confusion matrix of MobileNetV3_S, **(E)** is the confusion matrix of GhostNet, **(F)** is the confusion matrix of RepVggNet_B0, **(G)** is the confusion matrix of ConvNeXt_T, **(H)** is the confusion matrix of FasterNet_T0, and **(I)** is the confusion matrix of HPMobileNet.

As shown in **Figure 11** confusion matrices of different models, when comparing the confusion matrices of the other eight models, it can be noticed that each model performs differently on the classification task. Looking at the confusion matrix of HPMobileNet, we can see that the values on the diagonal line are relatively high, which means that the model performs well in correctly classifying mung bean seeds. The higher values on the diagonal line indicate that the model has a higher prediction accuracy for the corresponding category. Meanwhile, relatively low values on the off-diagonal line mean that the model misclassified samples into other categories less often. In contrast, the confusion matrices of the other eight models show different degrees of variation. The other models have lower values on the diagonal of the confusion matrix relative to HPMobileNet, suggesting that they are not as accurate as HPMobileNet on the classification task. Most of the models perform poorly on specific categories, as can be seen in the figure for the eighth category and the second category. However, the classification ability of HPMobileNet relative to the other models in the eighth category and the second category is not as good as the other models. Category and the second category, but HPMobileNet has significantly higher classification ability on the eighth category and the second category compared to the other models. This result shows that the improved model has a clear advantage in classification performance.

The precision, recall, and F1-scores of each network model on the eight categories were derived from the confusion matrix as shown in **Table 9**. The precision of HPMobileNet on each category was 0.937, 0.940, 0.922, 0.950, 0.943, 0.985, 0.936 and 0.912. The recall was 0.950, 0.891, 0.933, 0.961, 0.973, 0.948 and 0.912 respectively. 0.891, 0.933, 0.961, 0.956, 0.973, 0.948 and 0.912 respectively. The F1 scores are 0.943, 0.915, 0.927, 0.955, 0.949, 0.979, 0.942 and 0.912 respectively. The results show that the optimal results are obtained for each category of the HPMobileNet, which can be concluded that the network model proposed in this study shows excellent performance on several categories of mung bean seeds, especially on the GL07C and JL06C categories, where its accuracy and F1-score are significantly better than other classical models, showing its potential and advantages in complex tasks.

**Table 9:** Detailed comparison of Precision, Recall, and F1-score of different models in each category.

Model	Label	1	2	3	4	5	6	7	8
	Index	BLC	GL07C	GL13C	JL05C	JL09C	JL10C	JL11C	JL06C
ResNet50	P	0.891	0.892	0.869	0.910	0.893	0.939	0.844	0.791
	R	0.890	0.696	0.881	0.930	0.921	0.985	0.927	0.821
	F1	0.890	0.782	0.875	0.920	0.907	0.948	0.884	0.806
ResNeXt50	P	0.914	0.856	0.846	0.914	0.895	0.964	0.878	0.853
	R	0.925	0.785	0.919	0.924	0.945	0.931	0.904	0.795
	F1	0.919	0.819	0.881	0.919	0.919	0.947	0.891	0.823
Res2Net50	P	0.875	0.833	0.866	0.908	0.908	0.944	0.850	0.813
	R	0.907	0.748	0.893	0.887	0.895	0.965	0.891	0.807
	F1	0.891	0.788	0.879	0.897	0.901	0.954	0.870	0.810
MobileNetV3_S	P	0.895	0.862	0.902	0.946	0.874	0.932	0.875	0.846
	R	0.907	0.805	0.900	0.924	0.910	0.953	0.912	0.828
	F1	0.901	0.833	0.901	0.935	0.892	0.942	0.893	0.837
GhostNet	P	0.892	0.827	0.840	0.944	0.847	0.957	0.905	0.789
	R	0.892	0.825	0.886	0.882	0.950	0.928	0.862	0.791
	F1	0.892	0.826	0.862	0.912	0.896	0.942	0.883	0.790
RepVggNet_B0	P	0.923	0.835	0.895	0.916	0.871	0.965	0.876	0.873
	R	0.909	0.828	0.869	0.908	0.968	0.953	0.918	0.816
	F1	0.916	0.831	0.882	0.912	0.917	0.959	0.897	0.844
ConvNeXt_T	P	0.718	0.487	0.677	0.692	0.624	0.743	0.680	0.547
	R	0.729	0.417	0.519	0.824	0.851	0.889	0.669	0.381
	F1	0.723	0.449	0.588	0.752	0.720	0.809	0.674	0.449
FasterNet_T0	P	0.910	0.800	0.808	0.872	0.847	0.952	0.870	0.809
	R	0.839	0.755	0.924	0.919	0.933	0.936	0.881	0.707
	F1	0.873	0.777	0.862	0.895	0.888	0.944	0.875	0.755
**HPMobileNet**	**P**	**0.937**	**0.940**	**0.922**	**0.950**	**0.943**	**0.985**	**0.936**	**0.912**
	**R**	**0.950**	**0.891**	**0.933**	**0.961**	**0.956**	**0.973**	**0.948**	**0.912**
	**F1**	**0.943**	**0.915**	**0.927**	**0.955**	**0.949**	**0.979**	**0.942**	**0.912**

The bold values represent the results of the algorithm HPMobileNet proposed in this study.

### 3.3 Results of ablation experiments

To assess the effects of the DMS block, ECA block, and Mish activation function on model performance, ablation experiments were conducted in this study using MobileNetV2 as the base network model. As shown in **Table 10**, the results indicate that the performance of each metric model is improved when these three modules are integrated separately, thus improving its applicability to mung bean seed variety classification. In addition, the simultaneous integration of these modules further improved the accuracy of the model, while ensuring that the parameters and FLOPs of the model did not increase substantially, and the accuracy was indeed improved by 6.61% relative to the original model by only 0.136M and 0.003G, respectively. Overall, the comprehensive performance of the model has been substantially improved, providing more reliable and accurate classification results for mung bean seeds.

**Table 10:** Specific details of the results of the ablation experiment.

DMS block	Mish	ECA block	Acc (%)	P (Avg)	R (Avg)	F1 (Avg)	Params (M)	FLOPs (G)
			87.40	0.873	0.876	0.873	2.234	0.326
√			88.08	0.880	0.883	0.880	2.370	0.329
	√		89.66	0.896	0.897	0.896	2.234	0.326
		√	92.04	0.921	0.921	0.920	2.234	0.327
√	√		91.78	0.918	0.919	0.918	2.370	0.329
√		√	92.29	0.923	0.924	0.922	2.370	0.329
	√	√	92.75	0.927	0.928	0.927	2.234	0.327
**√**	**√**	**√**	**94.01**	**0.941**	**0.941**	**0.949**	**2.370**	**0.329**

The bold values represent the results of the algorithm HPMobileNet proposed in this study. √ represents the selected improved method.

### 3.4 Comparative results before and after model improvement

**Figure 12:**
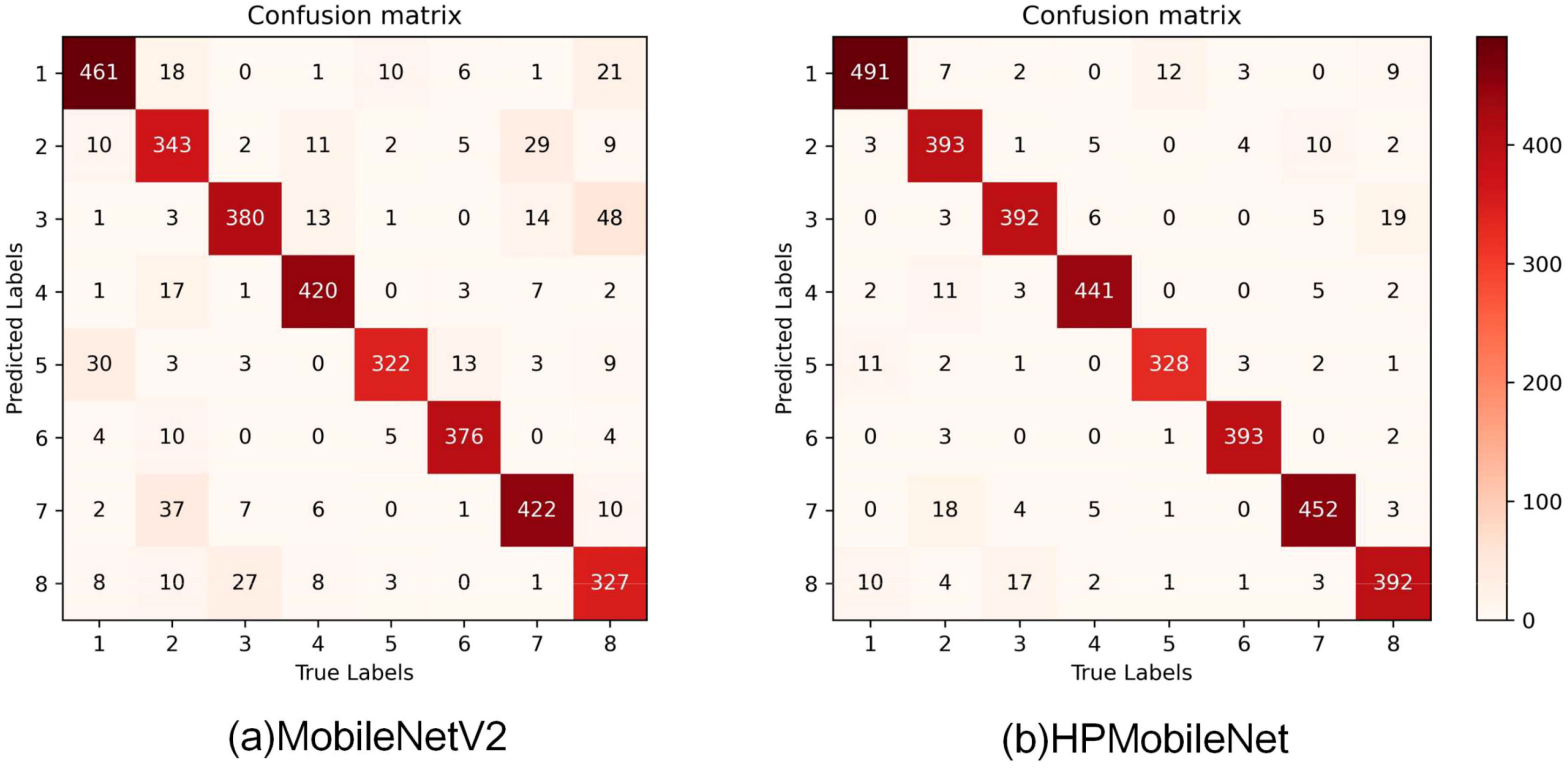
Visualization of the confusion matrix before and after model improvement, **(A)** is the confusion matrix of MobileNetV2, **(B)** is the confusion matrix of HPMobileNet.

To further validate the recognition ability of HPMobileNet and the original model, a comparison of the confusion matrices of the models before and after the improvement is given in this study. As shown in **Figure 12**, the improved network model effectively reduces the error rate of each category, especially significantly reduces the misclassification of the second category of mung bean seeds as the seventh category of mung bean seeds, the third category of mung bean seeds as the eighth category, and the fifth category of seeds as the first category of seeds. Macroscopically, the values on the diagonal have also improved significantly with darker colors, which indicates that the model’s recognition accuracy on all categories has improved significantly. In addition, the overall structure of the confusion matrix has become clearer, with a higher degree of differentiation between categories. In summary, the improved model can better extract features from mung bean seeds and shows more excellent performance, thus significantly reducing the recognition error rate and providing more reliable and accurate classification results for related applications.

The precision, recall, and F1-score of the model classification results can be obtained from the confusion matrix as shown in **Table 11**. The results show that the improved model improves all the metrics of the eight types of mung bean seeds compared to the original model. The precision of each type of mung bean seed grain was improved by 0.044, 0.105, 0.096, 0.019, 0.102, 0.043, 0.066, and 0.06. Meanwhile, the recall was improved by 0.058, 0.113, 0.028, 0.046, 0.017, 0.042, 0.063, and 0.152, respectively. In addition, the F1 scores were improved by 0.052, 0.11, 0.063, 0.032, 0.062, 0.043, 0.065 and 0.109. These results indicate that the improved network model exhibits better recognition performance in mung bean seed image classification.

**Table 11:** Comparative results of Precision, Recall, and F1-score for each category on the test set before and after model improvement.

Label	Name	P		R		F1	
		Before	After	Before	After	Before	After
1	BLC	0.890	0.934	0.892	0.950	0.891	0.943
2	GL07C	0.835	0.940	0.778	0.891	0.805	0.915
3	GL13C	0.826	0.922	0.905	0.933	0.864	0.927
4	JL05C	0.931	0.950	0.915	0.961	0.923	0.955
5	JL09C	0.841	0.943	0.939	0.956	0.887	0.949
6	JL10C	0.942	0.985	0.931	0.973	0.936	0.979
7	JL11C	0.870	0.936	0.885	0.948	0.877	0.942
8	JL06C	0.852	0.912	0.760	0.912	0.803	0.912

**Figure 13:**
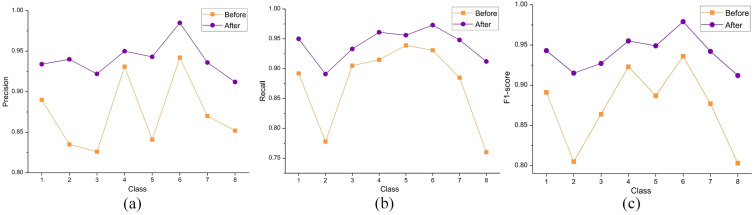
Visualized comparison results of Precision, Recall, and F1-score before and after model improvement, **(A)** denotes Precision comparison, **(B)** denotes Recall comparison, and **(C)** denotes F1-score comparison.

To better analyze the results before and after the improvement, this study visualized the data in the table, as shown in **Figure 13**, it can be seen more clearly that the precision difference between the model categories before the improvement is large, and the recognition ability of the model is poor. After the model improvement was completed, the precision and recall of each category reached a relatively high level, which led to the F1 scores also being greatly improved and showing a more balanced distribution. The recognition accuracies of all categories are improved compared with the pre-improvement period, and the differences in recognition accuracies between different categories are reduced, indicating that the overall performance of the model has been significantly improved, and the adaptability and generalization ability of the model in each category have been enhanced. For some categories with lower recognition accuracies before improvement (e.g., category 2 and category 8), the model gives more attention and optimization to improve their recognition accuracies significantly after improvement, so the overall performance of the model has been greatly improved.

**Figure 14:**
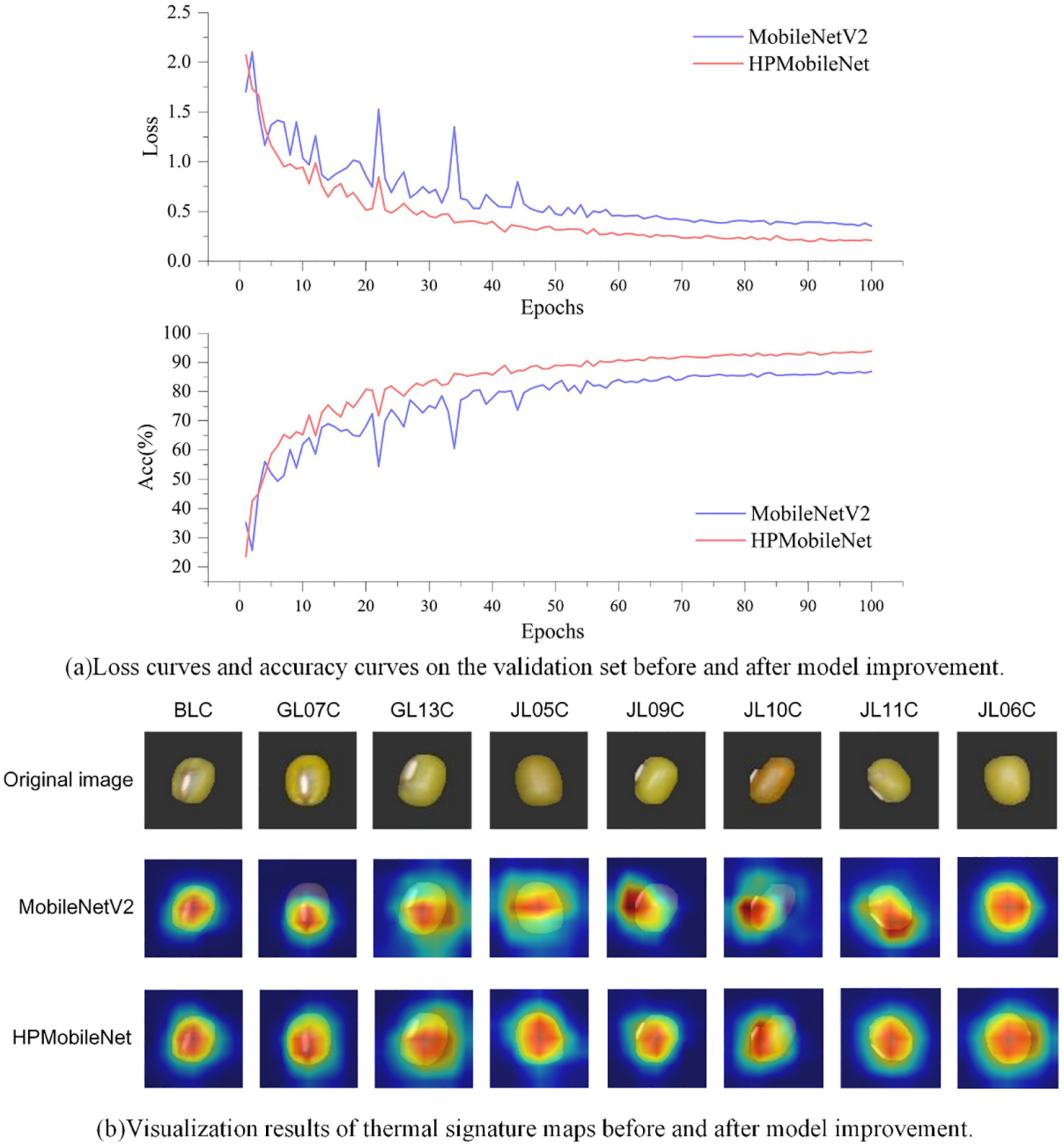
Comparison of accuracy curve, loss curve, and heat map before and after model improvement. **(A)** It shows the comparison of loss curves and accuracy curves on the validation set before and after model improvement. **(B)** It presents the heat feature maps before and after model improvement.

**Figure 14A** shows the loss curve and accuracy curve on the validation set before and after the model improvement. The accuracy curve before the model improvement still has large fluctuations at the beginning of the training period, but then the growth rate gradually slows down and stabilizes at the end of the training period, although the model can finally reach a relatively stable level of accuracy with the “95-Gradient”. Although the model can eventually reach a relatively stable level of accuracy with the “95-Gradient”, there are still large fluctuations in the pre-training period of the model, and there may be an overall risk of overfitting. In contrast, the accuracy curve of the improved model shows a more significant upward trend, with the accuracy increasing rapidly at the early stage of training, and then continuing to maintain a stable growth trend, finally reaching the highest accuracy level and stabilizing. Meanwhile, the loss curve of the pre-improved model fluctuates more in the early stage of training, although it gradually flattens out in the later stage of training, which indicates that the model encounters optimization difficulties in the training process, and it is difficult to further reduce the loss. In addition, the final value of the loss curve is relatively high, indicating that the model has limited fitting ability and may have overfitting or underfitting problems. The improved loss curve, on the other hand, shows a more desirable downward trend. In the early stage of training, the loss value decreases rapidly, then stabilizes gradually and fluctuates less in the pre-training period. This indicates that the improved model is more stable in the optimization process and can reduce the loss more effectively. Eventually, the lower value of the loss curve indicates that the fitting ability of the model has been significantly improved and can better adapt to the training data. By comparing the loss curves and accuracy curves before and after the improvement, we can see a significant improvement in the optimization ability and accuracy of the improved model.

To more intuitively analyze the effectiveness of the improved model in classifying mung bean seeds, this study used the visualization tool Grad-CAM (Selvaraju et al., 2017). Grad-CAM visualizes the image regions that the model focuses on during the prediction process by calculating the gradients of the feature mappings for the target class, multiplying these gradients with the feature mappings to obtain the weights, and ultimately generating a heat map. The original image is shown in the first row, while the second and third columns show the Grad-CAM mapped images before and after the model improvement. The spectrum from blue to red indicates the degree of contribution.

The experimental results are shown in **Figure 14B**. Before the model improvement, the model may focus more on the local features of the seeds, probably because the model does not have enough ability to extract the global features of the whole image. As a result, the heat map mainly focused on the local area of mung bean seeds, which caused the model to prioritize certain local features and ignore the overall features in the prediction process. However, after the model’s improvement, the model’s focus on channel information increased, enhancing its ability to capture global features. This enabled the model to better focus on the entire mung bean seed grain characteristics rather than just localized features during the prediction process. Thus, the comprehensive improvements in this study enhanced the feature extraction capability of the mung bean seed grain classification model, enabling it to more accurately localize valuable regions in mung bean seed grain images.

## 4 Discussion

The HPMobileNet proposed in this study is more efficient, less expensive, and more suitable for large-scale applications compared to traditional methods such as chemical and spectroscopic techniques. The accuracy is higher and the number of parameters is lower compared to classical and advanced deep learning algorithms. Despite the excellent experimental results obtained in this study, there are still some limitations that need to be verified in future studies.

Limitations of the dataset: The present study was conducted only on different varieties of mung bean and did not cover the same variety of mung bean grown in different regions or different varieties of mung bean grown in the same region. There are many effects of different geographic environments on the external phenotype of mung bean, such as moisture, temperature, light, and other factors, which are also affected by human factors, and more attention should be paid to the effects of objective factors on the external phenotype of mung bean in subsequent studies.

Limitations of crop types: This study focused only on the mung bean dataset, and the performance of the model on other crops has not been explored.

Limitations of model generalization: The ability of the model to generalize to different crops and environments remains to be fully verified.

In future studies, further parametric optimization of the model can be implemented in conjunction with the properties of MobileNetV4 (Qin et al., 2024). In data preprocessing, a new method for removing specular highlights from grayscale images (Xu et al., 2022) is applied to improve the algorithm’s ability to analyze and process images. Blind super-resolution (Xia et al., 2024) can also be achieved by meta-learning and Markov chain Monte Carlo simulation to make the original mung bean image more high-definition, which makes a corresponding basis for further analyzing the texture information of mung beans. The algorithms proposed in this study can be further embedded into target detection algorithms, such as YOLOv11 (Khanam and Hussain, 2024), to realize real-time monitoring of video streams with multiple targets for classification and identification. Meanwhile, the wireless charging flexible *in-situ* optical sensing (Zhang et al., 2024) can also be applied to the monitoring of mung beans in future research, which will in turn enable real-time data collection and thus improve the practical deployment of the model in smart agricultural systems.

## 5 Conclusion

In this study, the application of the improved network model HPMobileNet in mung bean seed classification is deeply explored. Important improvements are made in this study for the MobileNetV2 model, which introduces the efficient feature extraction module ECA block and the efficient residual block DMS block proposed in this study, respectively, and at the same time, the ReLU6 activation function is replaced with the Mish activation function. It is shown through extensive experimental validation that this integration not only drastically improves the accuracy of the model for mung bean seed classification, but also ensures that the FLOPs and parameters do not increase significantly, making HPMobileNet an ideal choice for resource-constrained environments. In the comparison experiments, the superiority of the learning rate dynamic adjustment strategy proposed in this study is verified, the performance of the ECA block and DMS block is verified, the efficiency of the Mish activation function is verified, and HPMobileNet is compared with eight other classical network models, and the results are shown through exhaustive performance evaluation and comparative analysis. HPMobileNet achieves optimal results for each integration, while HPMobileNet shows significant advantages in several key indicators, which are not only reflected in the accuracy of classification and recognition but also in its lightweight and high efficiency, which makes this network model have a broad application prospect in agricultural production, variety identification and other fields.

HPMobileNet cannot only be applied in the field of mung bean production but also be extended to the classification and quality inspection of other food and agricultural products, providing efficient and precise technical support for agricultural production. In summary, the rapid and accurate classification of different varieties of mung bean seeds based on HPMobileNet has significant application potential and practical value and provides new solutions and possibilities for automation and intelligence in the process of agricultural production.

## Data Availability

The original contributions presented in the study are included in the article/**Supplementary Material**. Further inquiries can be directed to the corresponding authors.

## References

[B1] Acquah C. Ohemeng-Boahen G. Power K. A. Tosh S. M. (2021). The effect of processing on bioactive compounds and nutritional qualities of pulses in meeting the sustainable development goal 2. Front. Sustain. Food Syst. 5, 681662. doi: 10.3389/fsufs.2021.681662

[B2] Barrio-Conde M. Zanella M. A. Aguiar-Perez J. M. Ruiz-Gonzalez R. Gomez-Gil J. (2023). A deep learning image system for classifying high oleic sunflower seed varieties. Sensors 23, 15. doi: 10.3390/s23052471 PMC10007379 36904675

[B3] Bellemare M. F. Lim S. (2018). In all shapes and colors: Varieties of contract farming. Appl. Econ. Perspect. Policy 40, 379–401. doi: 10.1093/aepp/ppy019

[B4] Bi C. Hu N. Zou Y. Zhang S. Xu S. Yu H. (2022). Development of deep learning methodology for maize seed variety recognition based on improved swin transformer. Agronomy 12, 1843. doi: 10.3390/agronomy12081843

[B5] Chen J. Kao S.-H. He H. Zhuo W. Wen S. Lee C.-H.. (2023). “Run, don’t walk: chasing higher FLOPS for faster neural networks,” in Proceedings of the IEEE/CVF conference on computer vision and pattern recognition. 12021–12031. Available online at: https://www.arxiv.org/abs/2303.03667v1.

[B6] Dahiya P. Linnemann A. Van Boekel M. Khetarpaul N. Grewal R. Nout M. (2015). Mung bean: Technological and nutritional potential. Crit. Rev. Food Sci. Nutr. 55, 670–688. doi: 10.1080/10408398.2012.671202 24915360

[B7] Ding X. Zhang X. Ma N. Han J. Ding G. Sun J. (2021). “Repvgg: Making vgg-style convnets great again,” in Proceedings of the IEEE/CVF conference on computer vision and pattern recognition. 13733–13742. Available online at: https://www.arxiv.org/abs/2101.03697v3.

[B8] Feng M. Q. Leung R. Y. Eckersley C. M. (2020). Non-contact vehicle weigh-in-motion using computer vision. Measurement 153, 107415. doi: 10.1016/j.measurement.2019.107415

[B9] Ganesan K. Xu B. Wellness H. (2018). A critical review on phytochemical profile and health promoting effects of mung bean (Vigna radiata). Food Sci. Human Wellnes 7, 11–33. doi: 10.1016/j.fshw.2017.11.002

[B10] Gao S.-H. Cheng M.-M. Zhao K. Zhang X.-Y. Yang M.-H. Torr P. (2019). Res2net: A new multi-scale backbone architecture. EEE Trans. Pattern Anal. Mach. Intell. 43, 652–662. doi: 10.1109/TPAMI.34 31484108

[B11] Ge Y. Song S. Yu S. Zhang X. Li X. (2024). Rice seed classification by hyperspectral imaging system: A real-world dataset and a credible algorithm. Comput. Electron. Agric. 219, 108776. doi: 10.1016/j.compag.2024.108776

[B12] Han K. Wang Y. Tian Q. Guo J. Xu C. Xu C. (2020). “Ghostnet: More features from cheap operations,” in Proceedings of the IEEE/CVF conference on computer vision and pattern recognition. 1580–1589. Available online at: https://www.arxiv.org/abs/1911.11907v2.

[B13] He K. Zhang X. Ren S. Sun J. (2016). “Deep residual learning for image recognition,” in Proceedings of the IEEE conference on computer vision and pattern recognition. 770–778. Available online at: https://www.arxiv.org/abs/1512.03385v1.

[B14] He L. Hu Q. Yu Y. Yu Y. Yu N. Chen Y. (2023). Discrimination of mung beans according to climate and growing region by untargeted metabolomics coupled with machine learning methods. Food Control 153, 109927. doi: 10.1016/j.foodcont.2023.109927

[B15] Howard A. Sandler M. Chu G. Chen L.-C. Chen B. Tan M.. (2019). “Searching for mobilenetv3,” in Proceedings of the IEEE/CVF international conference on computer vision. 1314–1324. Available online at: https://www.arxiv.org/abs/1905.02244v5.

[B16] Huang Z. Wang R. Cao Y. Zheng S. Teng Y. Wang F.. (2022). Deep learning based soybean seed classification. Comput. Electron. Agric. 202, 107393. doi: 10.1016/j.compag.2022.107393

[B17] Kanwal K. Zafar M. Khan A. M. Mahmood T. Abbas Q. Ozdemir F. A.. (2022). Implication of scanning electron microscopy and light microscopy for oil content determination and seed morphology of Verbenaceae. Microsc. Res. Tech 85, 789–798. doi: 10.1002/jemt.23950 34582087

[B18] Khanam R. Hussain M. (2024). YOLOv11: an overview of the key architectural enhancements. Arxiv.

[B19] Li Z. Keel S. Liu C. He Y. Meng W. Scheetz J.. (2018). An automated grading system for detection of vision-threatening referable diabetic retinopathy on the basis of color fundus photographs. Diabetes Care 41, 2509–2516. doi: 10.2337/dc18-0147 30275284

[B20] Li J. Xu F. Song S. Qi J. (2024). A maize seed variety identification method based on improving deep residual convolutional network. Front. Plant Sci. 15, 1382715. doi: 10.3389/fpls.2024.1382715 38803603 PMC11128617

[B21] Liu Z. Mao H. Wu C.-Y. Feichtenhofer C. Darrell T. Xie S. (2022). “A convnet for the 2020s,” in Proceedings of the IEEE/CVF conference on computer vision and pattern recognition. 11976–11986. Available online at: https://arxiv.org/abs/2201.03545.

[B22] Liyanage R. Kiramage C. Visvanathan R. Jayathilake C. Weththasinghe P. Bangamuwage R.. (2018). Hypolipidemic and hypoglycemic potential of raw, boiled, and sprouted mung beans (Vigna radiata L. Wilczek) in rats. Food Biochem. 42, e12457. doi: 10.1111/jfbc.2018.42.issue-1

[B23] Loddo A. Loddo M. Di Ruberto C. (2021). A novel deep learning based approach for seed image classification and retrieval. Comput. Electron. Agric. 187, 11. doi: 10.1016/j.compag.2021.106269

[B24] Ma S. Li Y. Peng Y. (2023). Spectroscopy and computer vision techniques for noninvasive analysis of legumes: A review. Comput. Electron. Agric. 206, 107695. doi: 10.1016/j.compag.2023.107695

[B25] Mehdizadeh S. A. Noshad M. Hojjati M. (2024). A modified sequential wavenumber selection-discriminant analysis with bayesian optimization strategy for detection and identification of chia seed oil adulteration using raman spectroscopy. Talanta. 277, 126439. doi: 10.1016/j.talanta.2024.126439 38897011

[B26] Misra D. (2019). Mish: A self regularized non-monotonic activation function.

[B27] Mukasa P. Wakholi C. Faqeerzada M. A. Amanah H. Z. Kim H. Joshi R.. (2022). Nondestructive discrimination of seedless from seeded watermelon seeds by using multivariate and deep learning image analysis. Comput. Electron. Agric. 194, 10. doi: 10.1016/j.compag.2022.106799

[B28] Naik N. K. Sethy P. K. Behera S. K. Amat R. J. (2024). A methodical analysis of deep learning techniques for detecting Indian lentils. J. Agric. Food Res. 15, 100943. doi: 10.1016/j.jafr.2023.100943

[B29] Nikolić Z. Đorđević V. Torbica A. Mikić A. (2012). Legumes seed storage proteins characterization by SDS-PAGE and Lab-on-a-Chip electrophoresis. J. Food Compos. Anal. 28, 75–80. doi: 10.1016/j.jfca.2012.08.005

[B30] Qin D. Leichner C. Delakis M. Fornoni M. Luo S. Yang F.. (2024). MobileNetV4 – universal models for the mobile ecosystem. Arxiv.

[B31] Sandler M. Howard A. Zhu M. Zhmoginov A. Chen L.-C. (2018). Mobilenetv2: Inverted residuals and linear bottlenecks. EEE/CVF Conference on Computer Vision and Pattern Recognition. 4510–4520. doi: 10.1109/CVPR.2018.00474

[B32] Selvaraju R. R. Cogswell M. Das A. Vedantam R. Parikh D. Batra D. (2017). Grad-cam: Visual explanations from deep networks via gradient-based localization. . Int. J. Comput. Vision. 128, 618–626. doi: 10.1109/ICCV.2017.74

[B33] Tarahi M. Abdolalizadeh L. Hedayati S. (2024). Mung bean protein isolate: Extraction, structure, physicochemical properties, modifications, and food? Applications 444, 138626. doi: 10.1016/j.foodchem.2024.138626 38309079

[B34] Wang Q. Wu B. Zhu P. Li P. Zuo W. Hu Q. (2020). “ECA-Net: Efficient channel attention for deep convolutional neural networks,” in Proceedings of the IEEE/CVF conference on computer vision and pattern recognition. 11534–11542. Available online at: https://www.arxiv.org/abs/1910.03151v4

[B35] Wu M. Li Y. Yuan Y. Li S. Song X. Yin J. (2023). Comparison of NIR and Raman spectra combined with chemometrics for the classification and quantification of mung beans (Vigna radiata L.) of different origins. Food Control 145, 109498. doi: 10.1016/j.foodcont.2022.109498

[B36] Xia C. Yang S. Huang M. Zhu Q. Guo Y. Qin J. (2019). Maize seed classification using hyperspectral image coupled with multi-linear discriminant analysis. IEEE Trans. Pattern Anal. Mach. Intell. 103, 103077. doi: 10.1016/j.infrared.2019.103077

[B37] Xia J. Y. Yang Z. X. Li S. X. Zhang S. H. Fu Y. W. Gunduz D. N.. (2024). Blind super-resolution via meta-learning and markov chain monte carlo simulation. IEEE Trans. Pattern Anal. Mach. Intell. 46, 8139–8156. doi: 10.1109/TPAMI.2024.3400041 38758618

[B38] Xie S. Girshick R. Dollár P. Tu Z. He K. (2017). Aggregated residual transformations for deep neural networks. IEEE Conference on Computer Vision and Pattern Recognition. 1492–1500. doi: 10.1109/CVPR.2017.634

[B39] Xie C. He Y. (2018). Modeling for mung bean variety classification using visible and near-infrared hyperspectral imaging. CVPR 11, 187–191. doi: 10.25165/j.ijabe.20181101.2655

[B40] Xu H. T. Li Q. Chen J. (2022). Highlight removal from A single grayscale image using attentive GAN. Appl. Artif. Intell. 36, 19. doi: 10.1080/08839514.2021.1988441

[B41] Yu H. L. Chen Z. Y. Song S. Z. Chen M. J. Yang C. L. (2024). Classification of rice seeds grown in different geographical environments: an approach based on improved residual networks. Agronomy-Basel 14, 24. doi: 10.3390/agronomy14061244

[B42] Yu Y. Wei X. Liu Y. Dong G. Hao C. Zhang J.. (2022). Identification and quantification of oligomeric proanthocyanidins, alkaloids, and flavonoids in lotus seeds: A potentially rich source of bioactive compounds. Food Chem. 379, 132124. doi: 10.1016/j.foodchem.2022.132124 35065486

[B43] Zhang R. H. Wang M. Zhu T. Y. Wan Z. Z. Chen X. J. Xiao X. Q. (2024). Wireless charging flexible *in-situ* optical sensing for food monitoring. Chem. Eng. J. 488, 8. doi: 10.1016/j.cej.2024.150808

[B44] Zhao Y. Wang Y. Wang L. Zhang D. J. F. Security E. (2020). Molecular identification of mung bean accessions (Vigna radiata L.) from Northeast China using capillary electrophoresis with fluorescence-labeled SSR markers. Food Energy Secur. 9. doi: 10.1002/fes3.182

[B45] Zhou L. Zhang C. Taha M. F. Wei X. He Y. Qiu Z.. (2020). Wheat kernel variety identification based on a large near-infrared spectral dataset and a novel deep learning-based feature selection method. Front. Plant. Sci. 11, 575810. doi: 10.3389/fpls.2020.575810 33240294 PMC7683420

