# Supplementary material for: Rapid and accurate classification of mung bean seeds based on HPMobileNet
Source: Front Plant Sci. 2025 Feb 13;15:1474906. doi: 10.3389/fpls.2024.1474906 (PMC11865048; doi:10.3389/fpls.2024.1474906)
Supplement: Supplementary file 1 [file DataSheet1.docx]

# Supplementary material

**Supplementary Table 1** Confusion matrix of the binary classification problem.

| **Confusion Matrix** | | **Actual Results** | |
| --- | --- | --- | --- |
|  |  | **Positive** | **Negative** |
| Forecast Results | Positive | TP | FP |
|  | Negative | FN | TN |

**Supplementary Table 2** Parameters for model training.

| **Set of Parameter** | **Value or Name** |
| --- | --- |
| Input Size | 224×224 |
| Batch size | 64 |
| Epoch | 100 |
| Learning rate base | 0.01 |
| Optimizer | SGD |
| Learning rate scheduler | ${LR}_{epoch}$=${LR}_{0}$×${decay rate}^{epoch//2}$ |
| Decay rate | 0.95 |

**Supplementary Table 3** Experimental environment configuration details.

| **Accessories** | **Name or Version** |
| --- | --- |
| Operating System | Windows10 |
| Development Framework | Pytorch1.12.1 |
| Development Language | Python3.8.3 |
| CUDA | 11.3 |
| CUDANN | 8302 |
| Anaconda | 2021.11-windows |
| GPU | NVIDIA Quadro RTX 8000(48G) |
| CPU | Intel(R) Xeon(R) Gold 6246R (3.4GHZ) |


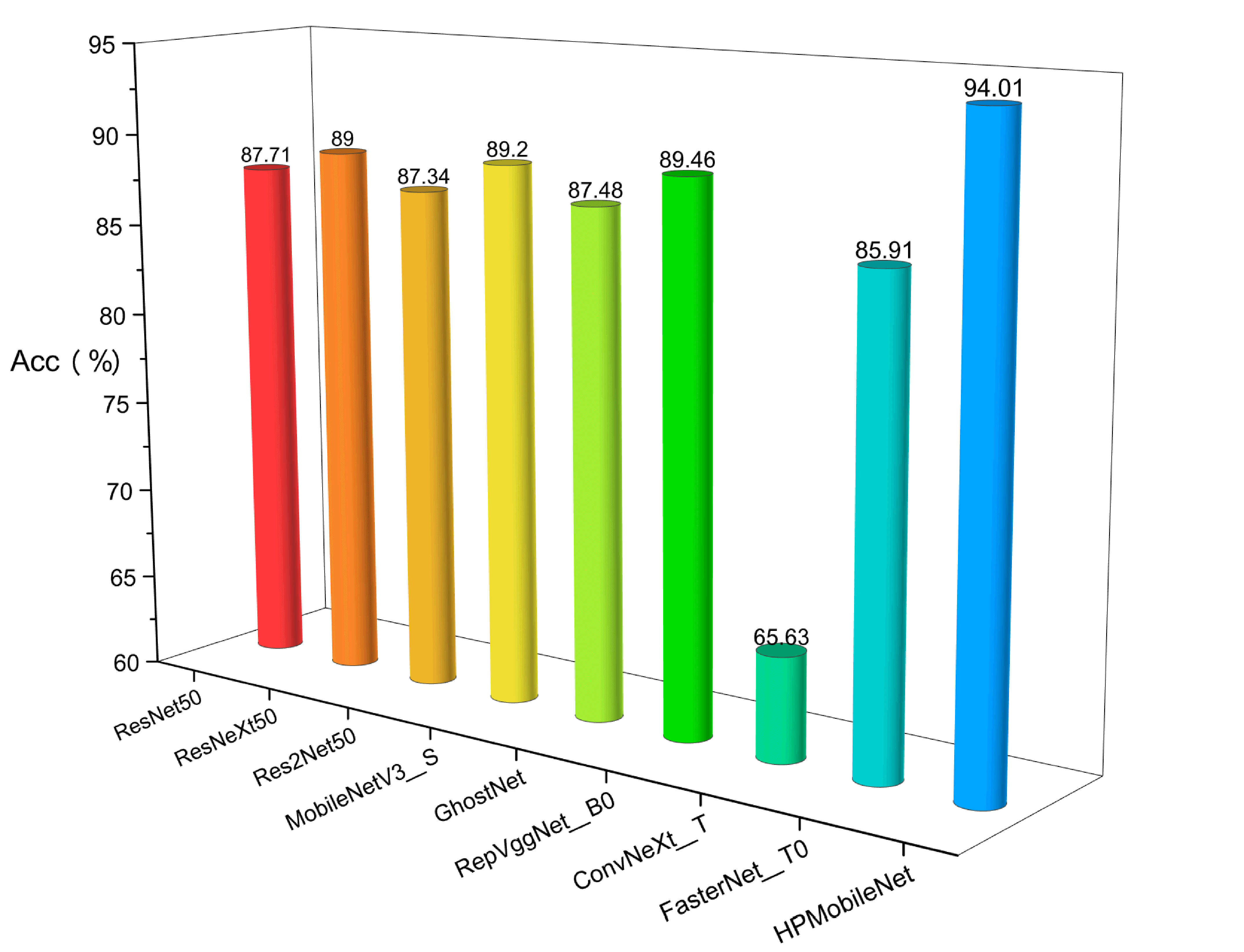


**Supplementary Figure 1** Accuracy visualization results for different models.
